# Pangenomic analyses in the cultivated grapevine confirm high genomic collinearity and extensive dispensable gene content likely involved in adaptation

**DOI:** 10.1093/g3journal/jkag168

**Published:** 2026-07-09

**Authors:** Baptiste Pierre, Roberto Bacilieri, Dominique This, Thierry Lacombe, Vincent Segura, Patrice This, Anne Mocoeur, Romain Villoutreix, Gautier Sarah

**Affiliations:** UMR AGAP Institute, Université de Montpellier, CIRAD, INRAE, Institut Agro Montpellier, Montpellier 34398, Occitanie, France; UMR AGAP Institute, Université de Montpellier, CIRAD, INRAE, Institut Agro Montpellier, Montpellier 34398, Occitanie, France; UMR AGAP Institute, Université de Montpellier, CIRAD, INRAE, Institut Agro Montpellier, Montpellier 34398, Occitanie, France; UMR AGAP Institute, Université de Montpellier, CIRAD, INRAE, Institut Agro Montpellier, Montpellier 34398, Occitanie, France; Geno-Vigne, IFV-INRAE-Institut Agro, Montpellier F-34398, Occitanie, France; UMR AGAP Institute, Université de Montpellier, CIRAD, INRAE, Institut Agro Montpellier, Montpellier 34398, Occitanie, France; Geno-Vigne, IFV-INRAE-Institut Agro, Montpellier F-34398, Occitanie, France; UMR AGAP Institute, Université de Montpellier, CIRAD, INRAE, Institut Agro Montpellier, Montpellier 34398, Occitanie, France; Unité expérimentale du domaine de Vassal, INRAE, Institut Agro Montpellier, Marseillan 34340, Occitanie, France; UMR AGAP Institute, Université de Montpellier, CIRAD, INRAE, Institut Agro Montpellier, Montpellier 34398, Occitanie, France; UMR AGAP Institute, Université de Montpellier, CIRAD, INRAE, Institut Agro Montpellier, Montpellier 34398, Occitanie, France

**Keywords:** Pangenome graph, *Vitis vinifera*, genome organization, GWAS, structural variants

## Abstract

Pangenomes have now been developed for several horticultural crops, yet the extent to which genome diversity in sequence and organization contribute to plant adaptation and major agronomic traits remains poorly understood. Here, we assembled the genomes of 9 cultivated grapevine varieties and compared the genomes of 15 cultivated grapevine varieties for variation in gene and TE content. We found that genomic collinearity is highly conserved among varieties. We still observed substantial variation across genomes. Notably, we identified across varieties 55,662 orthologous genes, of which 55.3% appears to be dispensable. Dispensable genes are enriched for functions related to adaptation to biotic and abiotic constraints, suggesting that they may play a role in adaptation. Comparing our results with a recently published study, we found substantial differences with ∼12.6% of the genes we classified as core genes being classified as dispensable genes in this other study. We then constructed a pangenome graph and used it to performed genome-wide association studies for 3 important traits in grapevine production, which allowed us to include large structural variants as markers in the analyses. We identified 32 loci that we did not detect when we used the PN40024 genome as a reference, 20 of which are newly reported associations. Overall, our results indicates that despite recent advances in characterizing plant pangenomes, current gene classification into core and dispensable gene categories should be taken with caution. They also highlight the value of incorporating structural variants into GWAS, to better characterize the genetic architecture of agronomic traits.

## Introduction

The cultivated grapevine (*Vitis vinifera* L. subsp. *vinifera*; shortened to *Vitis vinifera* hereafter) is an economically important crop. In 2024, it was cultivated on approximately 7.1 million hectares worldwide for the production of table grapes, wine or dry grapes (State of the World Vine and Wine Sector in 2024, 2025). To this day, around 6,000 varieties of grapevine, cultivated in a wide range of environments, have been inventoried (Wolkovich et al. 2018). Characterizing and leveraging the adaptive genetic diversity present within these varieties is critical, especially as viticulture faces increasing threats from climate change, with seasonal instability, late frosts, extreme heat waves, prolonged periods of rain or drought, and the emergence of new pests (Dry et al. 2019; Coupel-Ledru et al. 2024; van Leeuwen et al. 2024).

Recent studies started to characterize the genetic bases of adaptive traits such as phenology, drought or heat tolerance and disease resistance (Delfino et al. 2019; Coupel-Ledru et al. 2024; Guo et al. 2025). However, most of these studies used a single reference genome (usually a version of the PN40024 reference (Shi et al. 2023)) and may have missed a significant part of the genetic diversity present in cultivated grapevine (Schatz et al. 2014; Rey et al. 2016). Indeed, recent research in multiple animal and plant species suggests that the proportion of variable genes and sequences (ie genes and sequences not shared by all individuals in a species) can be very high in eukaryotes (Liu et al. 2020; Yan et al. 2023; Jayakodi et al. 2024), particularly in plants (Liu et al. 2020; Qiao et al. 2021; Liao et al. 2023; Yan et al. 2023; Jayakodi et al. 2024; Lian et al. 2024), reaching up to 50% of genes in soybean or pearl millet (Liu et al. 2020; Yan et al. 2023). This led to multiple pangenomic studies in plants, which aimed to capture a larger part of the diversity of sequences present within a species or population of interest, including both common and rare sequences (Bayer et al. 2020; Sherman and Salzberg 2020). Another suggestion of these recent pangenomic studies is that genome wide association studies (GWAS hereafter) realized with a pangenome graph as a reference may better characterize the genetic basis of traits than using a single reference genome (Kang et al. 2023; Liu et al. 2024).

In grapevines, a pangenome and 2 super-pangenome (ie pangenome produced at the genus levels (He et al. 2024)) have now been produced (Cochetel et al. 2023; Liu et al. 2024; Guo et al. 2025). Through comparative genome alignment and annotation, these studies revealed considerable variability in gene content and genome organization within the *Vitis* genus (Cochetel et al. 2023; Guo et al. 2025). For the cultivated grapevine, these studies revealed that only a minority of genes are shared by all genomes (ie core genes), with approximately 19% of genes shared (core genes) and more than 75% of genes not shared (variable genes) between genomes (Liu et al. 2024). These analyses also revealed the existence of several hundred thousand structural variants (SVs), of which approximately 31% overlap with coding regions, highlighting the high contribution of SVs in generating intraspecific genomic diversity (Liu et al. 2024). Despite these strong advances in providing the initial characterization of the pangenome of the cultivated grapevine, the varieties used to construct the pangenome in both studies (Liu et al. 2024; Guo et al. 2025) were not evenly sampled across the 3 genetic groups known to exist in the cultivated grapevine (Bacilieri et al. 2013; Nicolas et al. 2016), and they may have missed a substantial amount of sequence diversity in their pangenome. There is therefore an interest in replicating these analyses using a balanced sample to see if we obtain the same picture of pangenome organization, with similar core and variable gene sets identified and similar ratios of core and dispensable genes. This is the primary aim of this study, which we realized with comparative genome alignment and annotation approaches. The second aim of this study is to produce a pangenome graph and test if using it as a reference improves GWAS analyses, by allowing us to include large SV more easily into such analyses.

To do so, we sequenced and assembled the genomes of 9 cultivated grapevine varieties and selected 6 varieties whose genomes were publicly available, resulting in a balanced sample of 15 varieties across the 3 main genetic groups of the cultivated grapevine (Nicolas et al. 2016). We first analyzed the organization of the genomes across these varieties by assessing collinearity, variations in gene content, structure and biological function, and variation in transposable elements content. We then compared our results to past work (Liu et al. 2024). We then constructed a pangenome graph combining all 15 varieties and the PN40024 reference genome and studied its architecture and compared it with another pangenome graph developed in the same species (Liu et al. 2024). We then used this pangenome graph as a reference to genotype a panel of 277 varieties for SNPs and large structural variants, and then performed GWAS on 3 important grapevine agronomic traits: berry weight, ripening date, and shikimate concentration in musts. Finally, we compared our GWAS results with the literature to assess the value of this methodology in identifying novel loci.

## Materials and methods

### Plant materials

#### Overview of plant material

We used material coming from different sources (collections and publicly available genomes). The following subsections describe the material used for each analysis and its origin.

#### Pangenome individuals

We selected 15 varieties, along with the PN40024 reference (Shi et al. 2023) to conduct pangenome analyses and construct a pangenome graph. To ensure a balanced representation of the genetic diversity of the cultivated grapevine, we selected this set of varieties from the 3 main genetic groups present in the species (Table-East, Wine-East, and Wine-West genetic groups; Nicolas et al. 2016). Of these 15 varieties, we choose to sequence and assemble the genome of 9 varieties based on the availability of plant material within our collection. We selected varieties at random within each genetic group. For the remaining 6 varieties, we also selected varieties with published genomes at random within each genetic group to complete our balanced sampling. We also included the haplotype of the PN40024 reference to allow comparison with previously published studies.

More specifically, our sample comprise 9 newly sequenced and assembled varieties (“Alexandroouli”, “Blank blauer”, “Bou Khanzir noir”, “Camaraou noir”, “Corbeau”, “Morrastel”, “Opsimo Edessis”, “Pagadebiti”, and “Riminese”), corresponding to traditional varieties from different geographical origins, including notably Georgia, Morocco, France (south-western, Savoie, Corsica), Spain (la Rioja), Greece (Macedonia region), and Italy (Liguria), and 6 previously published genomes (“Chasselas”, “Ugni blanc” from Djari et al. 2024; “Manicure finger”, “Muscat Hamburg”, “Pinot noir”, “Sultanina” (syn “Thompson Seedless”) from Liu et al. 2024). These varieties belong to the following genetic groups: Wine-East group (“Blank blauer”, “Muscat Hamburg”, “Pagadebiti”, and “Riminese”), Table-East group (“Alexandroouli”, “Bou Khanzir noir”, “Manicure finger”, “Opsimo Edessis”, and “Thompson Seedless”), and Wine-West group (“Camaraou noir”, “Chasselas”, “Corbeau”, “Morrastel”, and “Pinot noir”). from

#### GWAS panel

To perform GWAS we used a panel of 277 varieties. This panel is described in previous work from our institute (Nicolas et al. 2016) but we provide a brief description of this material here. This panel is designed to maximize the amount of genetic diversity observed in the cultivated grapevine within a set of 277 individuals. These 277 varieties were selected from an initial set of 2,323 varieties (Laucou et al. 2011; Bacilieri et al. 2013; Nicolas et al. 2016), removing clones and first-degree relatives and selecting a core of 277 individuals to maximize the representation of the genetic diversity present in our collection (Vassal-Grape-BRC, France, https://eng-vassal.montpellier.hub.inrae.fr/). We provide the passport data for all 277 varieties of the panel that are also maintained within our collection (Vassal-Grape-BRC, Marseillan, France; Supplementary Table 1).

### Tissue collection for long-read, short-read, and RNA-seq sequencing

For varieties without already available data (genomes, short-reads, or RNA-seq) we collected fresh tissue from 2022 to 2024.

For long-read and short-read sequencing, we collected young leaves from plants in the Vassal collection. For RNA-seq sequencing we collected nonmature berries from the 9 newly sequenced and assembled varieties (“Alexandroouli”, “Blank blauer”, “Bou Khanzir noir”, “Camaraou noir”, “Corbeau”, “Morrastel”, “Opsimo Edessis”, “Pagadebiti”, and “Riminese”), as a part of a greenhouse experiment with contrasted irrigation performed in the phenoArch platform at Montpellier SupAgro (LEPSE, Montpellier, France).

### DNA extraction and library preparation for long-read sequencing

We extracted high molecular weight DNA (HMW) using the QIAGEN Genomic-tip 500/G kit. We then purified these extractions, fragmented them (average 20 kb), end-repaired, A-cut and ligated them to barcoded adapters to prepare SMRTbell(r) HiFi libraries. We size-selected the libraries (10–50 kb), and quantified them on a Qubit. We then pooled equimolarly these libraries to generate 3 final pools that we sequenced on a PacBio Revio system (INRAe Gentyane platform, Clermont-Ferrand, France). HiFi sequencing generated an average coverage of approximately 30–35× per variety.

### DNA extraction and library preparation for short read sequencing

Details on DNA extraction, library preparation, and sequencing can be found in a previous publication (Sarah et al. 2025) but we present a brief summary here.

We isolated genomic DNA using an INRAE protocol (Flutre et al. 2022). We then prepared Illumina short-read paired-end libraries using a PCR-free in-house protocol (INRAE ref. PEX-NGS-030). We multiplexed and sequenced these libraries on a NovaSeq 6000 (MGX platform, Montpellier, France). Theoretical short-reads coverage ranged from 13.7× to 60×.

### RNA extraction and library preparation for RNA-seq sequencing

We extracted total RNA from frozen berries (−80 °C) using a modified CTAB protocol. We weighed each berry, removed seeds and pedicels, and ground the tissue into a fine powder in liquid nitrogen using a GenoGrinder (1 min, 1,500 rpm) with metal beads. We lysed 300 mg of frozen powder in 2.5 ml of modified CTAB buffer (0.1 M MOPS, pH 7.8; 1.4 M NaCl; 1% PEG 4000; 10 mM EDTA; 2% CTAB; 2% β-mercaptoethanol) at 65 °C for 15 min with agitation. After centrifugation (20 min, 4,500 rpm, 4 °C), we extracted the supernatant with an equal volume of chloroform, incubated it on ice for 5 min, and centrifuged again. We precipitated RNA with 0.8 volumes of cold isopropanol (1 h at −20 °C), recovered it by centrifugation, washed it twice with 70% ethanol, resuspended it in 100 µl of ultrapure water, quantified it, and stored it at −20 °C.

We prepared stranded mRNA libraries from 1 µg of total RNA using the TruSeq Stranded mRNA LT kit (Illumina; INRAE internal number PEX-NGS-TruSeq RNA_SP). We purified poly(A) RNA with magnetic beads, fragmented it at 94 °C, and reverse-transcribed it using SuperScript II. We synthesized the second strand with dUTP incorporation and purified cDNA with AMPure XP beads. We repaired cDNA fragments, performed 3′ adenylation (30 min at 37 °C, then 5 min at 70 °C), and ligated indexed Illumina adapters (10 min at 30 °C). After 2 successive AMPure XP cleanups, we enriched libraries by PCR (15 cycles: 98 °C for 10 s, 60 °C for 30 s, 72 °C for 1 min) and purified them with AMPure XP beads. We assessed library size and quality on an Agilent Bioanalyzer (DNA 7500 kit) and quantified libraries by qPCR (LightCycler 480, Roche).

We multiplexed and sequenced libraries on a single lane of an S4 flow cell (150 bp paired-end; NovaSeq, Illumina). Quality control showed uniform read distribution, high base quality across cycles, and no major contamination. The sequencing effort was comparable for all 72 RNA-seq samples with the number of filtered reads per sample ranging from approximately 28 to 63 million, for a total of approximately 2.95 billion reads across the entire track, ensuring sufficient coverage for subsequent analyses.

### De novo assembly of pseudohaplotypes

Prior to assembly, we assessed long-read data quality using LongQC v1.2.0c (Fukasawa et al. 2020) with default settings. We then assembled the genomes of the 9 newly sequenced varieties into haplotype-phased contigs using hifiasm v0.19 (Cheng et al. 2021) as part of the ASM4PG workflow (Denni S et al. in prep), orchestrated with Snakemake v6.5.1 (Mölder et al. 2021). For each variety, we generated 2 haplotypes: a primary haplotype and an alternative haplotype. Because we did not use Hi-C data or genetic maps, we based phasing solely on the coverage and quality of PacBio HiFi reads. We ran hifiasm with default parameters, with particular attention to coverage depth (-l3 option) to ensure reliable assembly of heterozygous regions.

To reduce duplications and remove artificial haplotypes, we used Purge_dups (v1.2.5, Guan et al. 2020). We aligned reads to the assemblies using Minimap2 (v2.24, Li 2018, -x asm20) to generate PAF files, computed coverage and cutoff thresholds with pbcstat and calcuts with default settings, and removed duplicated regions using purge_dups (-2 -T cutoffs -c<pb.base.cov>), followed by sequence extraction with get_seqs with default settings. We evaluated the quality of the purged haplotype assemblies using different tools and statistics. We assessed haplotype completeness with BUSCO (v5.3.1, Manni et al. 2021) using the Eudicots_odb10 dataset, running in genome mode (-m genome) with multithreading (-c 20). We performed kmer–based analyses with KAT (v2.4.1, -m 21 -v, Mapleson et al. 2017), using Jellyfish-generated kmer files to produce spectra and spectra-cn plots. We detected telomeric sequences with FindTelomeres with default settings (https://github.com/JanaSperschneider/FindTelomeres.git) and conducted sequence formatting with GenomeTools (v1.5.9, default option). We also carried out complementary quality assessments with kmer completeness with Merqury with default option (v1.3, Rhie et al. 2020), and generated kmer databases using Meryl (v1.4.1, Rhie et al. 2020).

We then anchored and oriented haplotigs using the chromosomes of the PN40024 v5 genome as reference (Shi et al. 2023) with RagTag (v2.1.0, Alonge et al. 2019) and default options. We retained contigs that could not be placed on the PN40024 reference as unplaced scaffolds. We therefore obtained 2 pseudohaplotypes for each newly sequenced variety.

### Structural and functional gene annotation of pseudohaplotypes and comparison of gene content across varieties

#### Gene annotation

We started by structurally annotating genes in the 30 pseudohaplotypes we included in our pangenome. We did not reannotate genes for the PN40024 reference as it is already very well annotated and one of the inputs of our annotation pipeline. We combined 3 complementary approaches: annotation transfer, ab initio prediction, and ab initio prediction supported by RNA-seq evidence. We transferred the annotation of the PN40024 reference v5.1 (latest annotation for the PN40024 reference haplotype v5) using liftoff v1.6.3 with default parameters (Shumate and Salzberg 2021). We realized an ab initio prediction for each pseudohaplotype using Helixer v0.3.2 with the lineage land_plant_v0.3_a_0080.h5 (Holst et al. 2026). We realized an ab initio prediction supported by RNA-seq evidence using BRAKER3 v.3.0.8 (Gabriel et al. 2024) with default parameters using the combined RNA-seq data from berries 9 cultivated varieties and protein evidence from OrthoDB for Eukaryota as well as from the annotation of the version 5.1 of the PN40024 reference (Shi et al. 2023). We used AGAT v1.2.0 (Dainat 2022) to combine these 3 annotations, in the following order: annotation transfer, ab initio prediction supported by RNA-seq evidence, and ab initio prediction. We then cleaned the structural annotation with custom scripts to delete incomplete or incoherent genes (see Data and Scripts availability section).

To obtain genes functional annotation, we used Funcannotate (https://gitlab.cirad.fr/agap/cluster/snakemake/funcannotate). This workflow combines various tools, including HMMER v3.3.2 for searching Hidden Markov Model (HMM) profiles in sequence databases, SAMtools v1.14 for manipulating SAM/BAM/CRAM format files, SeqKit v2.0.0 for fast processing of FASTA/Q, Diamond v2.0.11 and NCBI-BLAST+v2.12.0 for protein sequence alignments, interproscan v5.67-99.0 and KofamSCan v1.3.0 for functional annotation based on protein families and KEGG orthology, Gffread v0.12.6 for GFF/GTF file processing, BUSCO v5.0.0 for gene set completeness assessment, Tabix (htslib v1.16.0) for tabulated file indexing, as well as R v4.1.0 and Bioperl (perllib v5.16.3) scripts for data processing and analysis. Using this pipeline, we performed functional annotation using the SwissProt and TrEMBL databases for similarity searches by Diamond, as well as HMMs defined for retrotransposons. We evaluated the completeness of our annotation using BUSCO v5.0.0, on sequences tagged as genes.

#### Gene clustering

Finally, for the analysis of gene content, we used GET_HOMOLOGUES-EST, a clustering tool based on nucleotide similarity of genes adapted for intraspecific pan-gene analyses (version 3.5.4, Contreras-Moreira and Vinuesa 2013). The main workflow comprised 3 scripts: get_homologues-est.pl, compare_cluster.pl, and parse_pangenome_matrix.pl. The get_homologues-est.pl script was used to generate orthologue clusters using the OMCL algorithm (-M). The parameters applied were as follows: minimum coverage of 80% of the shortest sequence in BLAST alignments (-C), maximum E-value of 1e-05 (-E), inclusion of all clustering (-t 0), with all other parameters set to their default values. The compare_clusters.pl script was used to compare across genomes and to produce the presence/absence matrix. Parse_pangenome_matrix.pl was used to analyze and transform this matrix in order to obtain the final gene content variation data. These last 2 scripts were used with their default parameters. The clusters were then filtered to distinguish conserved and variable genes. This workflow and these parameters ensure the reproducibility of the analysis and the generation of the pangene sets used for all gene content variation analyses (Contreras-Moreira and Vinuesa 2013).

We chose this approach rather than a direct integration of gene annotations into the pangenome graph, as current graph-based annotation tools still do not allow robust and standardized comparison of homology-based gene clusters across multiple genomes, whereas similarity-based clustering approaches remain the most widely validated and reproducible strategy for pangene inference.

#### Gene structure analysis

We imported, merged, and standardized GFF3 files for all pseudohaplotypes using R v4.5.3. We reconstructed gene characteristics by extracting attributes from the GFF3 files and aggregating genomic coordinates to calculate gene length, mean CDS length, and the number of exons per gene. We grouped genes into homology clusters, from which the core, soft-core, and variable categories were defined based on their distribution across the genomes of the 15 cultivated varieties, using presence/absence profiles. We performed statistical comparison between categories in R using an ANOVA followed by a Student-Newman-Keuls post-hoc test. We visualized the results using ggplot2 v4.0.1.

### Comparison of gene content and categorization between pangenomic studies

In order to compare the gene content across multiple studies, we applied the GET_HOMOLOGUES-EST tool to 2 distinct datasets: first on our dataset consisting of pseudohaplotypes from the 15 cultivated varieties and secondly on a combined dataset including these same varieties as well as those derived from the Grapepan v.1.0 pangenomic study (Liu et al. 2024) taking into account the cultivated grapevine varieties, enabling joint inference of homology-based gene clusters within a homogeneous framework and allowing comparison of gene repository richness and completeness between the 2 datasets. The resulting presence/absence matrices were then used for comparative analyses of gene content between datasets. We carried the analyses and visualizations in R v4.5.3 using the ggplot2 package v4.0.1 for generating plots, dplyr v1.1.4 and tidyr v1.3.1 for data manipulation, and ComplexUpset v1.3.3 for representing the intersections of homology-based gene clusters between datasets. The distributions of gene categories (core, soft-core, and variable) were visualized using merged barplots, enabling a comparison of the distribution of genomic compartments across the different datasets. In addition, we constructed Upset diagrams to represent the complex intersections of homology-based gene clusters across varieties and to highlight patterns of sharing and specificity within the pangene sets. These visualizations enabled us to summarize the overall structure of the gene content and to assess the similarities and differences in composition between the analyzed datasets.

### Transposable element annotation of pseudohaplotypes and comparison of transposable element content across varieties

#### Transposable element annotation

To annotate transposable elements (TEs hereafter), we applied a 4-step approach. We first built a database of repetitive sequences for each pseudohaplotype using RepeatModeler2 v2.0.6 BuildDatabase command (Flynn et al. 2020). We then ran RepeatModeler with the -database and -LTRstruct options to identify repetitive elements. We then curated and classified the resulting repeat libraries using MCHelper v1.7.0 (Orozco-Arias et al. 2024) with default parameters, leveraging the Viridiplantae BUSCO database (viridiplantae_odb10.hmm) to improve classification (Manni et al. 2021). We then used the curated repeat libraries as input for EDTA v1.10.0 (Ou et al. 2019) to perform structural annotation (--anno 1) and evaluation (--evaluate 1) of transposable elements. Finally, we combined TE annotations across all pseudohaplotypes using panEDTA (EDTA v1.10.0; Ou et al. 2024) to generate a consistent and comparable annotation among pseudohaplotypes. For visualization, we merged TE annotation outputs and generated graphical summaries with visualize_TE_annotations.qmd, facilitating interpretation of TE distribution patterns across varieties.

#### Transposable element-gene proximity analysis

When analyzing the overlap between orthologous genes and TE, a distance threshold of 5 kb was used to define the proximity between TEs and genes. This choice represents a compromise designed to capture the immediate regulatory environment of the genes, where the majority of functional effects associated with transposable elements are likely to occur (Gill et al. 2021).

### Construction of a pangenome graph

We constructed a pangenome graph with 31 pseudohaplotypes (15 cultivated varieties plus the PN40024 reference version 5). We constructed the graph using Minigraph-Cactus v2.9.0 (Hickey et al. 2024) through the cactus-pangenome pipeline, using the PN40024 genome as the reference sample (--reference). We selected PN40024 as the reference sample to ensure its path in the graph remained acyclic, forward-oriented, and suitable to generate a VCF, while remaining indexable for downstream mapping analyses. To determine the order in which pseudohaplotypes were added to the graph, we applied the barycenter method based on similarity distances computed with Mash (v2.3, command sketch and dist, Ondov et al. 2016), thereby minimizing the effect of insertion order on the final pangenome structure. We enabled several workflow options to generate all required outputs, including pangenome files in GBZ format (--gbz) and GFA format (--gfa), graph structures in ODGI format (--odgi), variant calling files (--vcf), mapping indexes (--giraffe), and graph visualizations (--viz and --draw). We measured the graph growth using the Gretl tool v0.1.1 (Vorbrugg et al. 2025) with default parameters.

### Genotyping (structural) variants in the association panel using the pangenome graph as a reference

We used the pangenome graph as a catalog to genotype variants present in our association panel of 277 varieties (Supplementary Table 1). Specifically, we used the Variation Graphs toolkit v.1.6 (VG toolkit, Hickey et al. 2020)). We indexed the pangenome graph using the sub commands vg autoindex for Giraffe mapping (-w giraffe), generating the GBZ file and minimizer indexes. We then aligned the short reads on the pangenome graph using vg giraffe with 20 threads, producing GAM alignment files. We extracted mapping statistics with vg stats, packed the read support with vg pack (using -s and -Q options), and called variants with vg call with default parameters and -a options, which is essential for merging VCFs from different samples. We removed 2 varieties from the original set of 279 varieties because they were either no longer present in the experiment parcel (ie replicates of the genotypes all died) or had been poorly sampled/sequenced due to incorrect identification in the field, which prevented reliable collection. Their exclusion helped to avoid introducing potential errors into the analysis (see Sarah et al. (2025) for details). We merged the remaining 277 samples into a single VCF file using the “merge all” command in BCFtools v1.17 (Danecek et al. 2021).

We then split the VCF file into 2 parts, 1 containing only the SNPs and another containing all other variants, including small variants and structural variants (>50pb). We did this separation using BCFtools v1.17 (Danecek et al. 2021). We then filtered the 2 matrices in similar ways using VCFtools (Danecek et al. 2011). Specifically, we kept variants with a minimum read depth of 10 and a minimum genotype quality of 30. We then retained sites with genetic information for at least 80% of individuals. After filtering, we transformed the VCFs into PLINK formatted files, and only retained bi-allelic variants. We then merged the 2 matrices mentioned above into a single matrix containing both SNPs and SVs (referred to in this study as the “pangenome matrix”). This PLINK matrix was then converted into a binary numerical format (0/1/2) using PLINK V2.00a3LM (Chang et al. 2015) for further analyses. Finally, we imputed missing data in these matrices with Beagle (Browning, Zhou and Browning 2018) using default parameters. For both small and large variation matrices, we solely applied a MAF ≥0.05 filter following biallelic filtering.

### Variant calling on the PN40024 reference

Using the short-read sequences of our 277 GWAS panel, we employed a GATK-based pipeline to obtain a variant matrix, using the PN40024 v5 genome as a reference (see Sarah et al. 2025). The complete workflow is available at: https://gitlab.cirad.fr/agap/cluster/snakemake/GATK.git.

Briefly, the pipeline cleans the reads with fastp v0.20.1, aligns the sequences to the reference using BWA-MEM2 v2.0, and removes duplicates with sambamba. We called genomic variants following GATK v4.5.0.0 best practices (HaplotypeCaller, GenomicsDBImport, GenotypeGVCFs). We filtered the resulting variant matrix using VCFtools, retaining only genotypes with GQ>30 and DP>10, then removing variants with more than 20% missing data. After filtering the final matrix contained 15,949,350 variants. After this initial filtering, we retained only biallelic variants. Additional filters were then applied to the genetic structure (MAF ≥0.05; Minor genotypic class frequencies ≥0.02; heterozygosity <0.6; *P*-value HWE >10–20), resulting in 3,452,492 variants. A final filtering by LD with Plink (indep-pairwise 50 5 0.99) yielded 1,608,936 variants, which were used for GWAS analyses.

### GWAS

#### Phenotypic data

We analyzed 3 agronomic traits in this study: shikimic acid concentration in musts, berry weight and maturity date. Phenotypic data were collected in 2011 and 2012 on the diversity panel, at the Domaine du Chapitre of Institut Agro Montpellier (Villeneuve-lès-Maguelone, France). Phenotypic measurements, statistical processing, broad sense heritability estimates and extraction of best linear unbiased predictors of genotypic values (BLUP) are described in detail in a previous study (Flutre et al. 2022).

#### Genotypic data

We performed GWAS using the 2 genotypic matrices described in the previous sections: (1) a combined matrix of SNPs and structural variants from the pangenome graph and (2) SNPs and small indels detected using the PN40024 reference genome. In order to reduce redundancy between highly correlated markers and to limit potential biases caused by linkage disequilibrium on association signals and PIP estimates, we applied LD filtering prior to all GWAS analyses using PLINK (indep-pairwise 50 5 0.99).

#### GWAS models

For each matrix, we tested 4 association models: GLM, MLM, and FarmCPU (implemented in rMVP) (Price et al. 2006; Yu et al. 2006; Liu et al. 2016; Yin et al. 2021), and BSLMM (implemented in GEMMA) (Zhou et al. 2013).

For the MLM and FarmCPU models, we estimated genomic relationships (kinship) directly from the pangenome matrix using the VanRaden method implemented in rMVP (Yin et al. 2021). We controlled population structure by including the first 5 principal components analysis in the GLM and the first 3 principal components in the MLM and FarmCPU models. For FarmCPU, we set the binning method to *static* and used the default maximum number of iterations. For BSLMM, we calculated genome-wide kinship matrices using LDAK v5.2. Specifically, we first split the pangenome matrix into sections to calculate marker weights using the –cut-weights and –calc-weights options, then we grouped the weights together using –join-weights. We then calculated the kinship matrix directly (--calc-kins-direct) using the weighted, with a power parameter of 0.25 and raw output (--kinship-raw YES). We did not include any additional covariates in the models. We used the resulting kinship matrices as input for GEMMA BSLMM, ensuring that population structure and kinship were correctly accounted for.

For the BSLMM analyses in GEMMA, we extracted posterior estimates for several parameters: the proportion of phenotypic variance explained by all genotyped markers (PVE), the proportion of PVE attributable to major effect loci (PGE), the number of major effect loci, and the posterior inclusion probability (PIP) for each marker. These parameters allow the characterization of genetic effects and loci with major effects, while controlling for linkage disequilibrium and kinship.

#### Robustness of associations, interpretations, and prediction of variant effects

To assess the robustness of associations identified with BSLMM, we compared its results with those obtained using complementary GWAS methods described previously. An association was considered robust if it was significant in BSLMM and at least 1 other method.

For interpretation, variant level associations were grouped into loci based on genomic proximity and linkage disequilibrium (LD), calculated while accounting for the kinship matrix using the LDcorSV package (v1.3.4). This approach enabled the identification of independent signals and avoiding redundancy from perfectly linked markers, between which BSLMM may randomly switch as they explain the same variance in the chain.

Given the high density of markers in the GLM, MLM, and FarmCPU models, we applied a false discovery rate (FDR) threshold of 5% (Benjamini-Hochberg correction) to account for multiple testing. For visualization in the Manhattan plots, the corresponding significance threshold was defined as the maximum value of −log10(P) among SNPs with adjusted *q*-values <0.05. Bonferroni thresholds are also shown in the figures for visual reference, whilst significance decisions are based on the FDR approach. To select reliable loci from the GWAS results, we cross-referenced the results of the 4 association models and identified loci based on the linkage disequilibrium calculated per chromosome (available here: https://doi.org/10.57745/CKD4H5). Only loci supported by at least 2 models were retained.

Finally, we annotated SNPs and other genetic variations in the selected locus regions using SnpEff (Cingolani et al. 2012), enabling the identification of candidate genes and functional variants potentially involved in trait variation. This combination of local LD analysis and functional annotation ensures that the reported loci are both statistically robust and biologically relevant.

### Phylogenetic inference and pseudohaplotypes collinearity analysis

Using the SNPs from our pangenome VCF generated by vg deconstruct, we constructed a phylogeny using the vcf2phylip v2.8 (Ortiz 2019) and RAxML-ng v1.0.2 tools (Stamatakis 2014). The alignment included 16 taxa (15 varieties and PN40024) and 1.2 million biallelic variants. We inferred phylogenetic relationships using the GTR+G substitution model with 25 parsimony-based starting trees and 25 random starting trees, followed by a constrained search under the GTR+I+G4 substitution model with 100 random starting trees. We then used the resulting phylogeny to define the order of genome insertions in the comparative genomics analysis.

We then performed pairwise genomic comparisons among 31 pseudohaplotypes using SyRI v1.7.0 (Goel et al. 2019) on alignments generated with Minimap2 (asm5; Li 2018) or NUCmer (Marçais et al. 2018). We split FASTA files by chromosome and processed them with Samtools or MUMmer utilities (delta-filter and show-coords) to identify structural rearrangements, including inversions, translocations, duplications, and deletions.

We visualized intra-chromosomal rearrangements using plotsr v1.1.1 (Goel and Schneeberger 2022), with genome-specific configuration files automatically generated by the workflow (https://gitlab.cirad.fr/agap/workflows/syri_combination.git). We conducted analyses across all 19 chromosomes, generating separate plots for Minimap2- and NUCmer-based alignments.

## Results

### Genome sequencing and assembly of 9 cultivated grapevine varieties

**Fig. 1.**
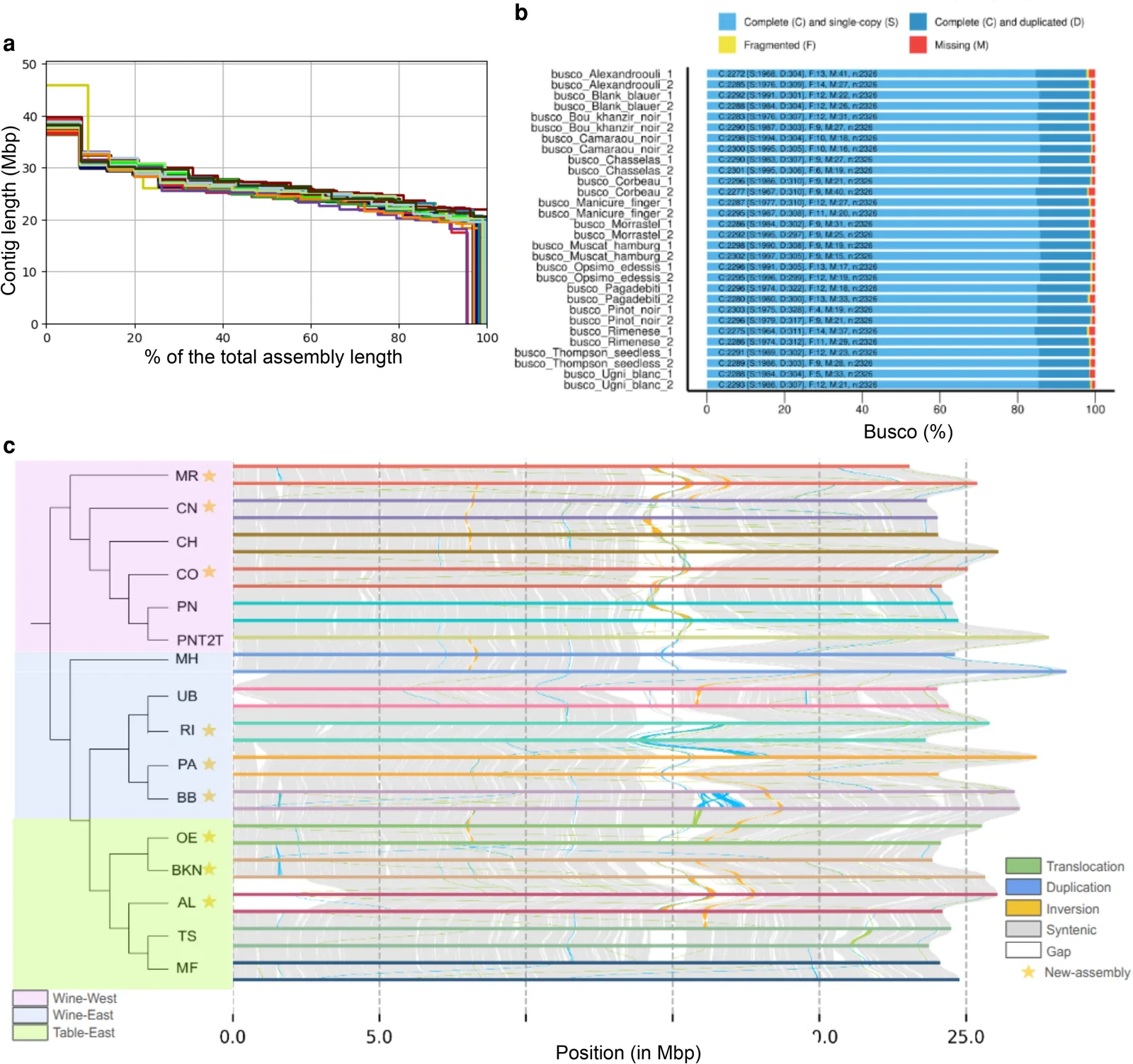
Genome assembly statistics and collinearity estimates across 15 cultivated grapevine varieties and the PN40024 reference. a) Continuity of genomic assemblies of 15 cultivated grapevines varieties, and the PN40024 reference. Continuity is assessed using NG(x) curves. NG(x) corresponds to the length of the contig such that x% of the expected genome size is covered by contigs of equal or greater length. The similarity of NG(x) profiles between varieties indicates high continuity and homogeneity in the quality of the assemblies. b) Assessment of assembly completeness using BUSCO scores, indicating a high proportion of complete genes, mostly in single copies, and very few fragmented or missing genes. c) Genome collinearity along chromosome 1. Genomes are ordered according to their genetic group of origin (Wine-West, Wine-East, Table-East). Colored segments indicate syntenic regions, duplications, inversions, translocations and gaps, highlighting an overall collinearity in chromosomal organization, punctuated by local structural variants. Stars indicate newly generated assemblies. MR: “Morrastel”, CN: “Camaraou noir”, CH: “Chasselas”, CO: “Corbeau”, PN: “Pinot noir”, PNT2T: PN40024, MH: “Muscat Hamburg”, UB: “Ugni blanc”, RI: “Riminese”, PA: “Pagadebiti”, BB: “Blank blauer”, OE: “Opsimo edessis”, BKN: “Bou Khanzir noir”, AL: “Alexandroouli”, TS: “Sultanina”, MF: “Manicure finger”.

We produced PacBio HiFi sequences for 9 cultivated varieties (“Alexandroouli”, “Blank blauer”, “Bou Khanzir noir”, “Camaraou noir”, “Corbeau”, “Morrastel”, “Opsimo Edessis”, “Pagadebiti”, and “Riminese”) and used these data to produce diploid assemblies. The final size of pseudohaplotypes ranged from 481.4 Mb to 521.5 Mb, with N50 ranging from 24.3 Mb to 27.1 Mb (Fig. 1a, Supplementary Table 2). We used a kmer approach to evaluate the sequence completeness of the pseudohaplotypes for each variety and found that the combination of pseudohaplotypes captures, on average, more than 99% of the kmer diversity observed in the filtered PacBio reads (Supplementary Fig. 1). We then ran a BUSCO analysis for each pseudohaplotype and found an average completeness score of 98.1% (min. 97.5%, max. 98.5%; Fig. 1b). Average assembly error, estimated by QV, remains very low (1 error per 4 billion bases). All these statistics confirm that the pseudohaplotypes we produced capture a large amount of the sequence diversity present in the filtered PacBio reads.

### Estimation of genomic collinearity across 15 cultivated grapevine varieties and the PN40024 reference

We then collected 6 publicly available assemblies to make our set of 15 cultivated varieties and assessed chromosomal collinearity across all 15 varieties. We found that chromosome arms were generally highly collinear across the cultivated varieties and the PN40024 reference. Nevertheless, we detected several rearrangements, large enough to be visible in the SyRi plot (including insertions, deletions, inversions, duplications, and translocations), although such events appear to be relatively infrequent. Overall, the assemblies are predominantly collinear across all cultivated varieties and the PN40024 reference, with rearrangements mainly located in specific chromosomal regions, particularly subtelomeric and centromeric regions (Fig. 1c, Supplementary Fig. 2).

### Variation in gene content across 15 cultivated grapevine varieties

**Fig. 2.**
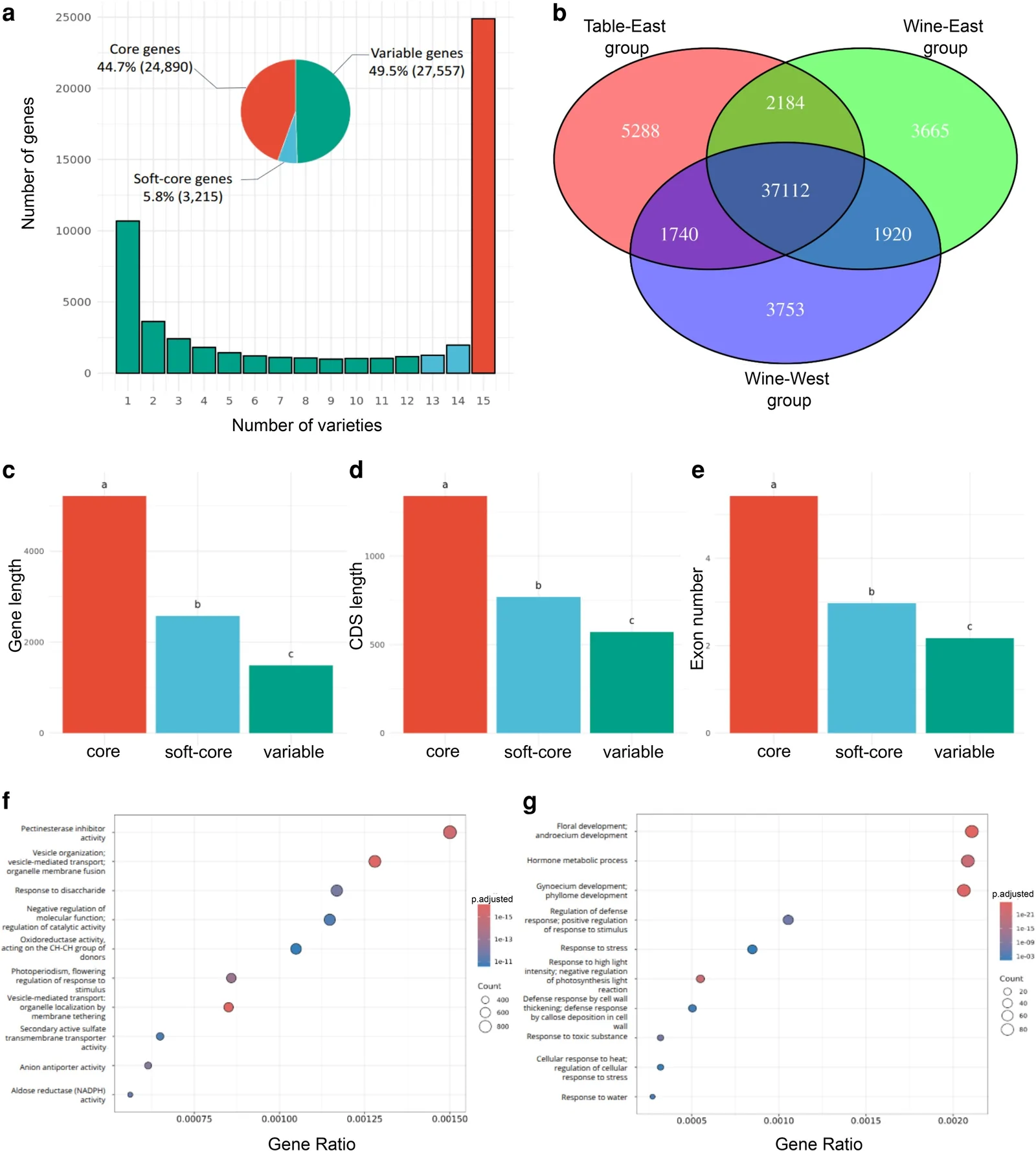
Variation in gene content across 15 cultivated grapevine varieties and differences in structure and function between pangene sets. a) Variation in gene content across 15 grapevine varieties. The histogram shows the number of ortholog genes present in 1 to 15 varieties. The pie chart shows the proportion of core, soft-core (shared by 14 or 13 varieties) and variable genes (shared by less than 13 varieties) (b) Venn diagram showing the overlap of genes between the 3 genetic groups of *V. vinifera* (Table-East, Wine-West, Wine-East). c) Average gene length for core, soft-core and variable genes (d) Average length of CDSs for core, soft-core and variable genes. e) Average exon number for core, soft-core and variable genes. We used the student Newman Keuls test for multiple comparisons to detect significant differences between groups; different lowercase letters indicate significant differences at the *P* < 0.05 threshold. f) Gene ontology (GO) enrichment analyses of core genes. The bubble chart shows the significantly enriched biological terms, with the *x*-axis representing the ratio of enriched genes over the total number of genes (GeneRatio), the size of the bubbles representing the number of enriched genes and the color the level of significance (*P*.adjust). g) GO enrichment analysis for variable genes. The bubble chart shows the significantly enriched biological terms, with the *x*-axis representing the ratio of enriched genes over the total number of genes (GeneRatio), the size of the bubbles representing the number of enriched genes and the color the level of significance (*P*.adjust).

First, we predicted an average of 48,322 genes per pseudohaplotype (min. 47,534; max. 49,342). We observed high BUSCO scores for sequences annotated as genes (97.7–99%) and a high proportion of genes with known predicted biological function (mean 83.35%), confirming the quality and completeness of our annotations (Fig. 2a; Supplementary Table 3).

We then analyzed the variation in gene content across the 15 cultivated varieties, considering the combined information from both pseudohaplotypes for each variety, using classical gene annotation comparison tools. We excluded the PN40024 reference from these analyses because it is known to result from multiple generations of selfing which have yielded a loss of a significant proportion of heterozygosity, genes, and sequences (Shi et al. 2023; Peng et al. 2025). We clustered genes into ortholog gene clusters using GET_HOMOLOGUES_EST. In this study, a pangene set corresponds to the set of orthologous gene clusters detected across the 15 cultivated varieties. This analysis identified 55,662 orthologous gene clusters (ie equivalent to orthologous genes; orthologous genes hereafter). Among orthologous genes, 24,890 (44.7%) are present in all 15 cultivated varieties and represent the core gene set, 3,215 (5.8%) are present in 13 or 14 varieties and represent the soft-core gene set, and 27,557 (49.5%) are present in 12 varieties or less and represent the dispensable and private gene sets (we merged these 2 categories into the single category of variable gene set hereafter; Fig. 2a). The structural differences between these orthologous gene clusters are marked: on average, core genes have significantly greater gene lengths, longer CDSs and a higher number of exons than soft-core and variable genes (Fig. 2c–e; Student-Newman-Keuls test: all pairwise comparison *P* < 0.05). This shows that gene architecture varies across the different compartments of the pan-gene sets, potentially reflecting differences in evolutionary constraints, levels of gene conservation and structural stability between these categories. The functional difference between gene sets also appeared clearly when we performed gene ontology enrichment analyses. The core gene set is enriched in genes with essential cellular functions, including primary metabolism, photosynthesis, basic enzymatic activities, and abscisic acid binding (Fig. 2f, Supplementary Table 4). The soft-core and variable gene sets are enriched in genes involved in secondary metabolism pathways, cell repair, and responses to biotic and abiotic stresses (Fig. 2g, Supplementary Fig. 3, Supplementary Table 5).

We then looked at the repartitions of genes among the 3 genetic groups of the cultivated grapevine (ie Table-East, Wine-East, and Wine-West). To do so, we grouped varieties into the genetic group they belong to (see Fig. 1c) and looked at orthologous genes shared at the genetic group level. We found that 37,112 (66.7%) orthologous genes are shared by all 3 groups, 5,844 (10.5%) are shared by 2 groups and 12,706 (22.8%) ortholog clusters are specific to a single genetic group (Fig. 2b). The Table-East group seems to contain the highest number of specific genes (5288). It is interesting to note that the Wine-East group shares more genes with the Wine-West group (1,920) and the Table-East group (2,184), while Wine-West and Table-East appear more distant with less genes shared among them (1,740), suggesting that Wine-East occupies an intermediate position between the other 2 groups, not only genetically, but perhaps also in terms of usage and geographical distribution.

### Comparison of gene content and categorization between pangenomic studies

We compared our pangene set and the pangene set from Liu and colleagues (Liu et al. 2024) considering only the cultivated grapevine (Grapepan hereafter). This analysis revealed a wide set of shared core genes between studies, with 15,290 genes classified as core in both studies (Supplementary Figs. 4 and 5). However, a significant number of genes were not detected or classified in the same category between studies. For example, 3,930 core genes in our pangene set are not identified in Grapepan, while 1,182 core genes from Grapepan are not identified in our dataset (Supplementary Fig. 4). Significant differences in category assignment are also observed between the core, soft-core, and variable categories. For example, 3,142 core genes in our study (∼12.6% of the core genes of our study) are classified as variables in Grapepan, and 549 variable genes (∼2% of the variable genes in our study) are classified as core in Grapepan (Supplementary Fig. 5). More broadly, a substantial proportion of genes is not shared between the 2 pangene sets, with 47% of the genes in our dataset absent from Grapepan, and 41% of Grapepan genes absent from our dataset. These results indicate that beyond the conserved core, a significant portion of the differences observed between pangene sets stems both from the use of different annotation methods and from differences in sampling, which influence categorization of genes into different categories (core, soft-core and variable genes).

### Variation in transposable element content among 15 cultivated grapevine varieties

**Fig. 3.**
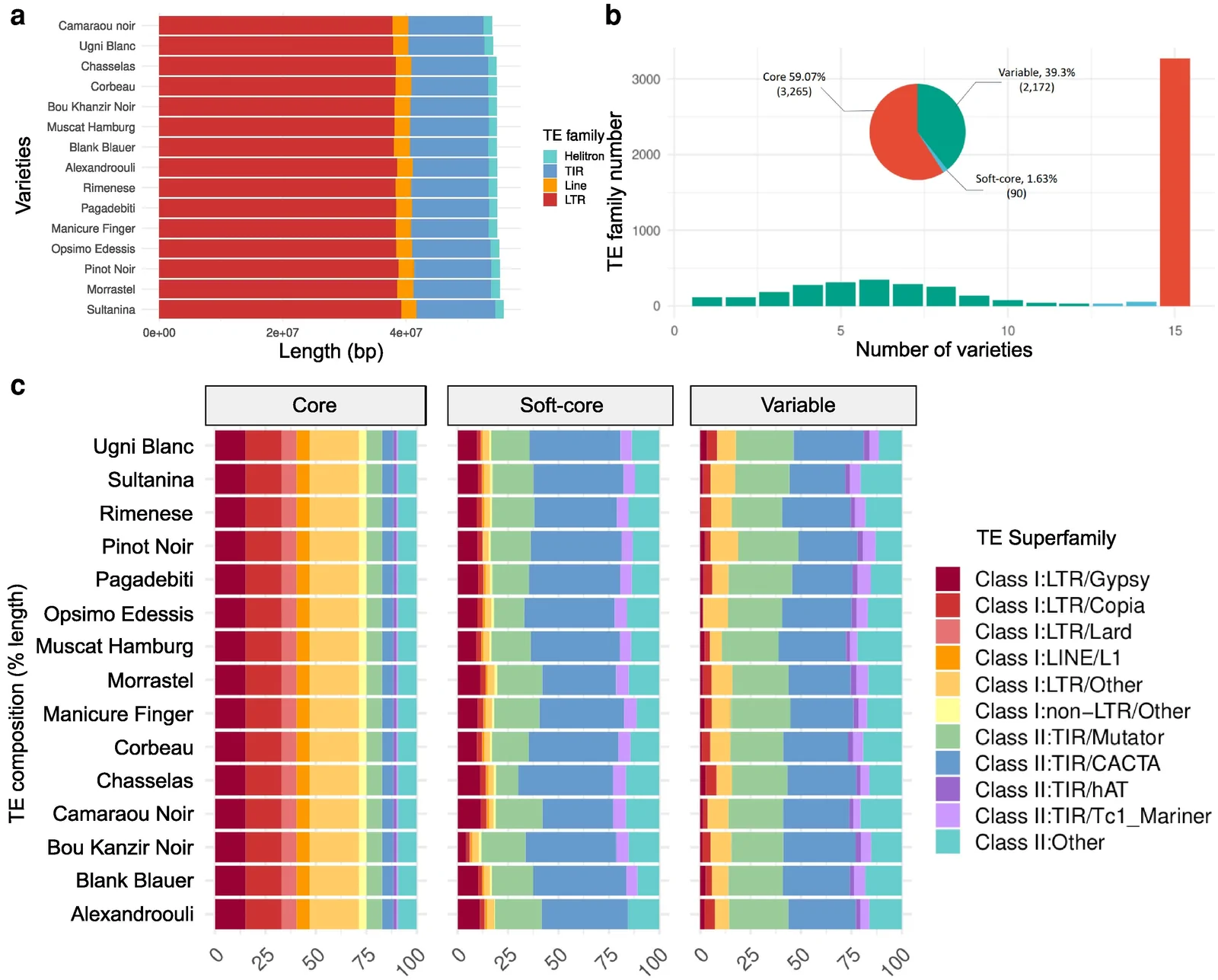
Diversity, distribution and composition of panTE families across 15 grapevine varieties. a) Total length of repeated regions per TE superfamilies for each variety. Colors correspond to the main TE superfamilies (Helitron, Line, TIR and LTR). The bar indicates the average proportion of repeat length covered by each TE type across all varieties. b) Distribution of shared TE families across 15 grapevine varieties. The barplot indicates the number of panTE families present in 1 to 15 varieties. The pie chart distinguishes between the core, soft-core, and variable panTEs categories. c) Distribution of the relative abundance of the different TE super-families for each grapevine variety. On panel is displayed for the core, soft-core, and variable TE compartment. Each line corresponds to a variety; different colors indicate different TE superfamilies.

We independently annotated TEs in each pseudohaplotype using panEDTA (Ou et al. 2024). We identified 5,527 panTE families (a set of similar TEs grouped by homology and structural features, panTE families hereafter; Ou et al. 2024) and 112,904 unclassified low-copy TE (Supplementary Table 6), covering an average of 52.36% of the pseudohaplotype sequences. A large majority of detected TE copies were incomplete or fragmented (89.2%) reflecting an accumulation and a progressive degradation of their sequences over time. Among the nonfragmented TE copies, LTRs dominate (Long Terminal Repeats, 68.8%), followed by TIRs and classical DNA transposons (Terminal Inverted Repeats, 22.5%) and, to a lesser extent LINEs (Long Interspersed Nuclear Elements, 4.6%) (Fig. 3a).

We then estimated which panTE families are shared between our grapevine varieties by evaluating their presence or absence using panEDTA, based on sequence similarity and genomic localization. We found that 59.07% of panTE families are present in all varieties (core TEs), 1.63% are present in 13 or 14 varieties (soft-core TEs), and 39.2% are present in 12 varieties or less (variable TEs) (Fig. 3b). We grouped nonfragmented TE copies into superfamilies (Wicker et al. 2007), and estimated the relative abundance of each superfamily within these different genomic compartments (core, soft-core and variable panTE). We found that core panTEs are predominantly composed of class I TEs superfamilies, while soft-core and variable panTEs are mainly composed of class II TE superfamilies (Fig. 3c). TE superfamily composition is, however, relatively homogeneous among varieties.

In order to assess the potential influence of TEs on gene regulation, we analyzed the proximity between TEs and genes across all pseudohaplotypes. We considered a TE to be close to a gene when it is located within a ±5 kb window around the gene coding region (Table 1; Supplementary Table 7a). Depending on the variety considered, between 13,392 and 18,987 TEs are found within a ±5 kb window around genes, with an average of 0.59 TEs per gene (Table 1). On average, 31.30% of genes (30.29–32.14%) have at least 1 TE in this window. When we reduce the window size to ±1 kb, this proportion drops to 11.1% (10.45–11.66%, Supplementary Table 7a), indicating that, while TEs are frequently located near genes, very adjacent localization remains relatively rare. However, the overall trends remain consistent across varieties when both distance thresholds are used. The proportion of genes with at least 1 TE located within a ±5 kb window is lower for the core gene set (29%) than for the soft-core gene set (37%) and the variable gene set (42%) (Supplementary Table 7b). Genes with at least 1 TE located within a ±5 kb window are significantly enriched for functions related to translation (Ribosome and tRNA biogenesis), DNA repair, primary and secondary metabolism, developmental processes, and responses to environmental stimuli such as nutrients, hormones, and stress (Supplementary Table 8). Interestingly, some genes with at least 1 TE located within a ±5 kb window are associated with well characterized phenotypic traits. Notably, we detected the *VvMYBA1* gene in our analyses, which is associated with the white berry phenotype (Kobayashi et al. 2004). This phenotype results from the insertion of the transposable element Gret1 at the *VvMYBA1* locus in white-berried varieties such as “Chasselas”, “Opsimo Edessis”, “Pagadebiti”, “Ugni Blanc”, and “Riminèse”.

**Table 1.** Summary statistics of gene and TEs annotations and their overlap.

	Min	Max	Average	Median
Total functionally annotated genes per genome	31,719	43,269	40,278	42,107
TEs within ±5 kb of a gene	13,932	18,987	17,476	18,247
Average of TEs within ±5 kb of a gene	0.55	0.63	0.59	0.59
genes with ≥1 TE in ±5 kb	9,659	13,834	12,623	13,255
% genes with ≥1 TE in ±5 kb	30.29	32.14	31.30	31.4

TEs are considered as overlapping a gene when they are located within 5 kb or 1 kb of the upstream and downstream region of a gene.

### Construction of a pangenome graph with 15 cultivated grapevine varieties and the PN40024 reference

We then constructed a pangenome graph, using the 30 pseudohaplotypes from the 15 cultivated varieties and the PN40024 haplotype reference. The PN40024 haplotype was used as an anchor path to harmonize coordinates, and assign nodes to pangenome graph compartments (core sequences, soft-core sequences, variable sequences). By retaining PN40024 as a reference, we ensure the stability of the coordinates, while the other pseudohaplotypes introduce additional genetic variation into the pangenome. The pangenome graph we constructed contains 67,459,944 nodes (pangenomes sequence units, representing distinct genomic fragments) and 92,316,889 edges (connections between nodes, indicating the order and continuity of sequences in pseudohaplotypes), for 1 Gb of genomic sequence, which is more than twice the size of the PN40024 haplotype (467 Mbp). Among these nodes, 29,689,054 are absent from the PN40024 reference path, and represent additional sequences present in other pseudohaplotypes but absent from the PN40024 reference (Supplementary Table 9a). The nonreference nodes in the pangenome graph have an average size of 12–13 bp and an average degree of 3 (computed as the sum of incoming and outgoing edges per node) along the variety paths (Supplementary Table 9b). Although the majority of sequence variants correspond to short variants (SNPs and Indels) that are generally specific to individual pseudohaplotypes, the graph also contains larger structural variants. The low connectivity of most nodes suggests that alternative paths remain simple and sparsely branched, giving the pangenome graph an overall topology of low complexity.

**Fig. 4.**
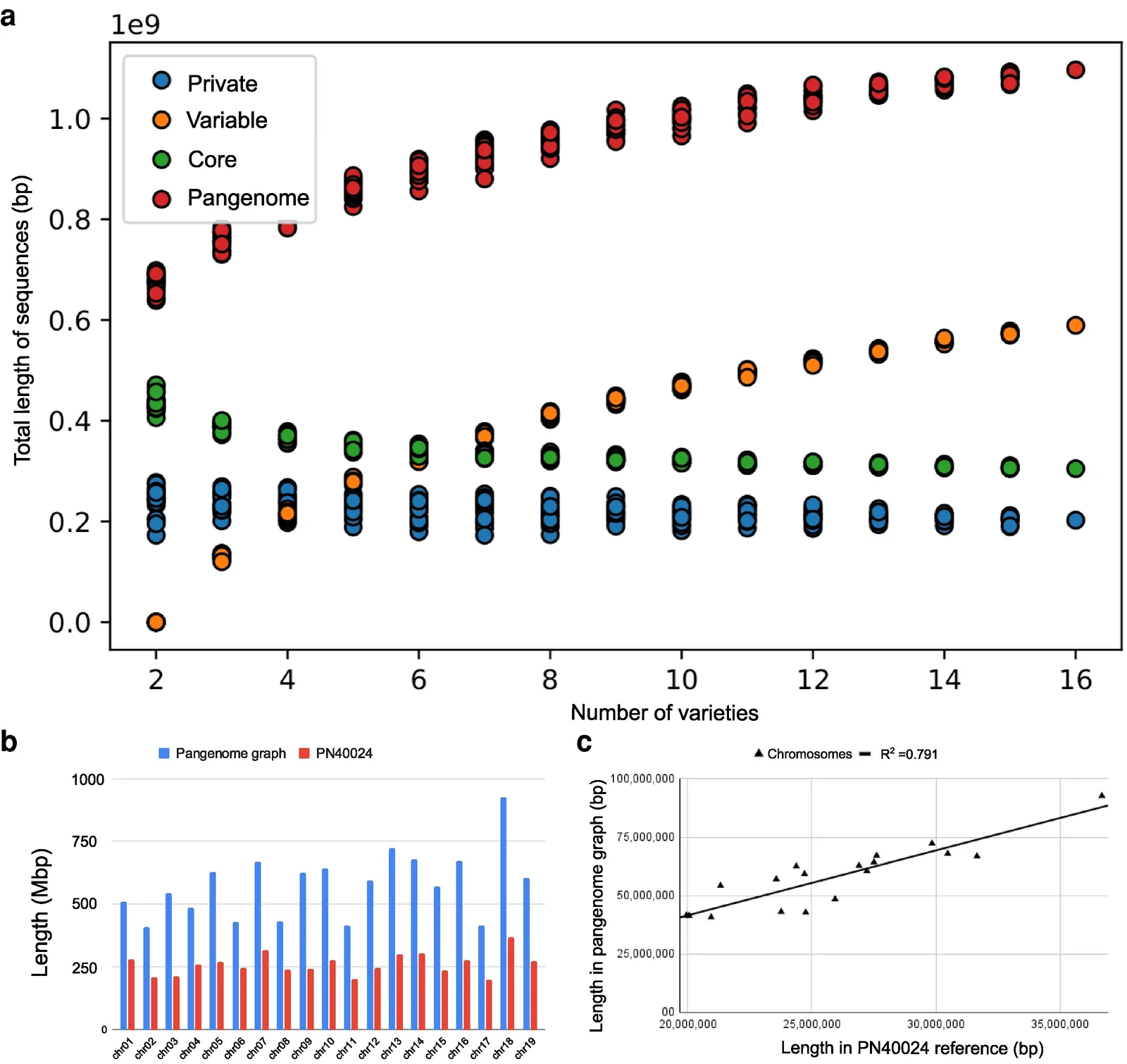
Growth of the pangenome graph and comparison of chromosome sizes. a) Pangenome graph sequence length growth curve, showing the increase in the pangenome graph sequence length as more genomes (ie 2 pseudohaplotypes per genome except for PN40024) are incorporated. The compartments are distinguished by color: Core sequences in green, variable sequences in yellow and private sequences in blue; the sum of these forms the complete pangenome graph, colored in red. The “variable” and “private” compartments are shown separately: the variable compartment increases cumulatively as genomes are added, whilst the private compartment may decrease when certain sequences that were initially private become shared, illustrating the gradual contribution of each genome. b) Barplot showing the size of chromosomes in base pairs in the pangenome graph (blue) and in the PN40024 reference (red). The lengths indicated for the pangenomes are calculated from nodes associated with each chromosome. c) Correlation between the length of chromosomes in reference PN40024 and the length of chromosomes in the pangenome. Each dot represents a chromosome.

We then analyzed the structure of the pangenome graph using the Gretl toolkit (Vorbrugg et al. 2025). On average, the addition of the genome (ie 2 pseudohaplotypes) of any cultivated variety adds 257,722 additional nodes, corresponding to 6.85 Mb of sequences (min. 4.39 Mb max. 10.52 Mb; Supplementary Table 9c). On average, the genome of each cultivated variety covers 83.15% (min. 82.3%, max. 84.5%) of the total sequence length of the pangenome graph (eg “Riminese”: 82.3%, “Pinot Noir”: 84.5%) (Supplementary Table 9b), which indicates that the majority of sequences present in the graph are shared, while a significant proportion (∼16.8%) of the sequences varies among cultivated varieties. The growth in sequence length of the pangenome graph and the decrease in size of core sequences tends to plateau and stabilize after the addition of 12 or more grapevine varieties (Fig. 4a).

The distribution of alternative nodes among chromosomes shows that all chromosomes have alternative sequences, with a cumulative length ranging from 18.1 Mb (chromosome 6) to 55.9 Mb (chromosome 18), for an average of 31.7 Mb per chromosome (Fig. 4b, Supplementary Table 9a). We also tested the correlation between the length of chromosomes in the PN40024 reference and in the pangenome, and observed a strong correlation between these 2 estimates (*R*^2^ = 0.79, *P-value* = 1.67 × 10^−6^, Fig. 4c, Supplementary Table 9a).

### Characterization of genetic variants captured in the pangenome graph

We then characterized the type and number of variants (SNPs, indels, translocations, duplications, inversions) captured by the pangenome graph. To do this, we deconstructed the pangenome graph with VG deconstruct (Liao et al. 2023) and the PN40024 genome as reference. Genetic variants located in regions absent from the PN40024 reference haplotype sequence could not be positioned and represented in the PN40024 coordinate space and are therefore not considered in the following analyses of this section (but they are still characterized and used in the GWAS analyses presented in the following section; See M&M for details). In total, we identified 13,344,565 variants, 92.78% of which are single nucleotide polymorphism (SNPs) or multiple nucleotide polymorphisms (MNPs), 13.09% are small indels (<50 bp) and 2.33% are other types of structural variants (>50 bp; insertions, deletions, inversions, translocations; SVs hereafter). Moreover, 13% of the genetic variants identified are multiallelic (Table 2).

**Table 2.** Summary of genetic variants in the pangenome graph.

Variable	Value	Percentage
Number of varieties	15	–
Number of genetic variants	13,344,565	100
Number of no-ALTs	0	0
Number of SNPs	12,380,581	92.78
Including MNPs	911,073	6.83
Number of indels	1,746,936	13.09
Number of others structural variants	310,496	2.33
Number of multiallelic variants	1,817,462	13.62
Including multiallelic SNPs	538,167	4.03

These statistics were generated with bcftools stats. The term “multiallelic” refers to sites with more than 2 alleles (ie 2 or more alternative alleles at a given position). The sum of SNPs, indels and other variant types may exceed the total number of variants, as multiallelic sites may include several types of variants (eg an SNP and an insertion/deletion) and are therefore counted in multiple categories. The “other structural variants” category includes complex substitutions, structural variants (SVs) and complex rearrangements, ie variants that are not classified as SNPs, MNPs or indels.

ALT, no-alternative allele; SNP, single nucleotide polymorphism; MNP, multiple nucleotide polymorphism.

After excluding SNPs and MNPs, we focused exclusively on small indels and SVs, using a positional approach in which each genomic locus is counted only once. Using this conservative approach, we retained a total of 1,481,737 genetic variants. Among these, 87,577 variants (approximately 5.9%) are classified as structural variants greater than 50 bp, while 1,394,160 variants (approximately 94.1%) are small genetic variants of 50 bp or less. For multiallelic sites, classification was performed by considering the largest size difference between the reference allele and all alternative alleles. Thus, each locus is counted as a single variant, regardless of the number of alternative alleles present, which may influence the distribution of variants around the 50 bp threshold. In contrast, the values reported in Table 2 are counts per allele and per category (as implemented by bcftools stats), meaning that multiallelic sites and variants belonging to multiple categories may be counted multiple times. This difference in methodology explains the difference observed between the 2 approaches.

We then analyzed the distribution of SVs along chromosomes and observed a strong depletion of SVs in centromeric regions (Supplementary Figs. 6 and 7). However, this observation could be due to the high structural complexity and richness in repeated sequences of these regions, which complicates the detection of both SNPs and SVs. We also observed that SVs more frequently localize in genomic regions with few or no SNPs (Supplementary Fig. 7). However, this observation should be interpreted with caution, as VG deconstruct only takes into account top-level variants along reference paths, excluding nested genetic variants (LV > 0) and thus potentially biases this estimate, giving the impression that SNPs are virtually absent in regions where only SVs are present.

We then examined the overlap of the 87,577 SVs positioned in the coordinates of the PN40024 reference with gene and TEs annotated on this reference haplotype sequence. We found that 33.47% (ie 29,316) of SVs overlapped with at least 1 gene and 70.19% (ie 61,473) of SVs overlapped with a TE.

### Genotyping known structural variants in a diversity panel to improve GWAS

**Fig. 5.**
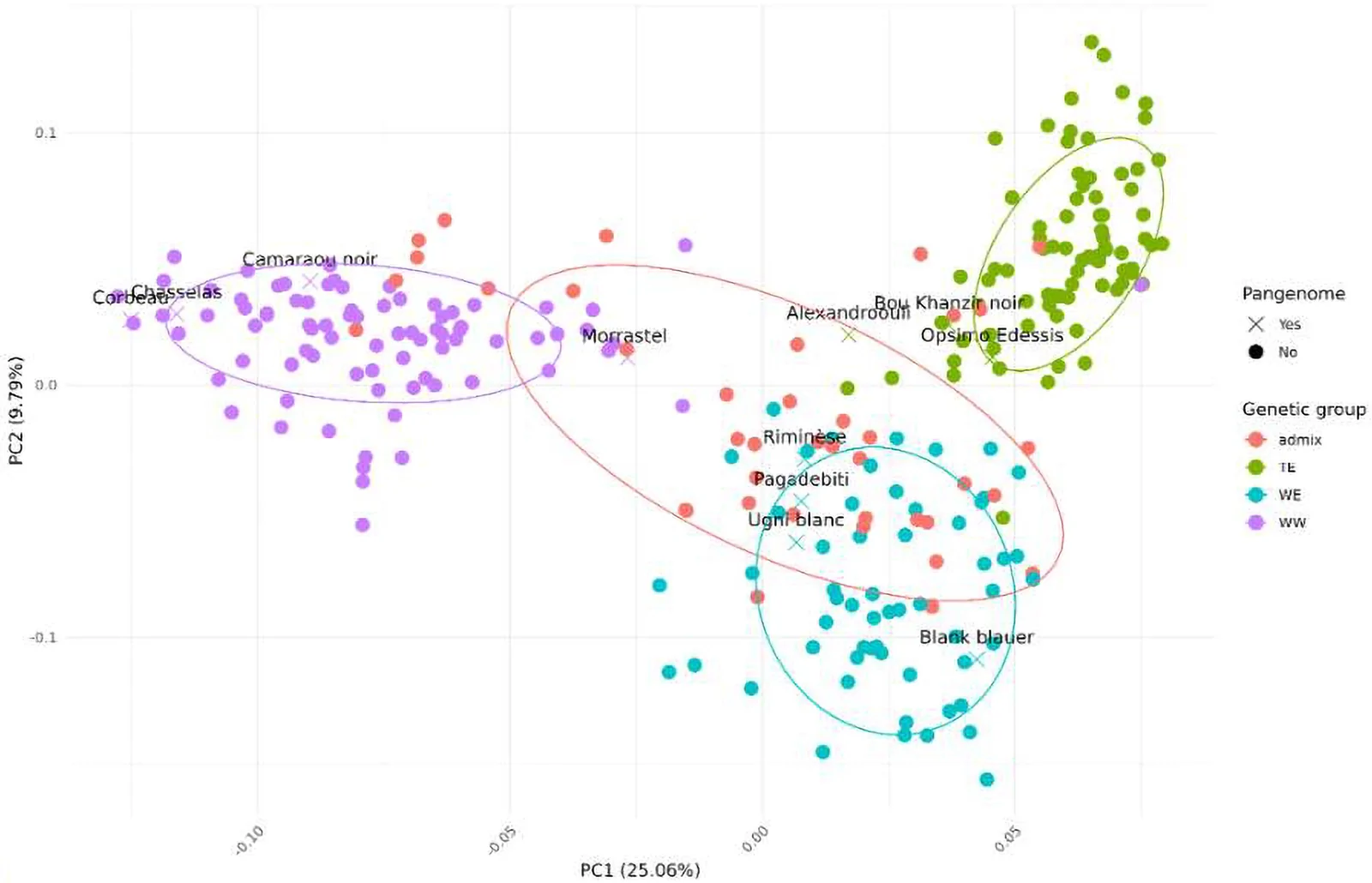
Principal component analysis of genome-wide variants in a GWAS panel of 277 grapevine varieties. We performed the PCA using ∼1.2 M markers genotyped from the pangenome graph. We recover the 3 main genetic groups previously identified using 20 SSR markers, reflecting both usage (Table *vs* Wine) and geographic origin (East *vs* West) (Nicolas et al. 2016). Grapevine varieties used to construct the pangenome graph (also included in the GWAS panel) are highlighted. Genetic groups are represented with different colors. Pink: Wine-West, blue: Wine-East, green: Table-East (Supplementary Fig. 8).

We assessed whether genotyping our association panel using the pangenome graph as reference, which includes many structural variants and sequences absent from the PN40024 reference, improves the characterization of the genetic basis and architecture of agronomic traits, notably by identifying loci missed by “traditional” GWAS using a single genome as reference. To this end, we genotyped our association panel of 277 varieties for variants using the pangenome graph as a catalog of genetic variants. After filtering, we retained ∼1.2 million biallelic variants (SNPs, small indels and structural variants) in all our subsequent analyses. We first verified that the variants captured within our graph allows us to capture a substantial proportion of the genetic diversity present in the cultivated grapevine. We performed a principal component analysis on these variants and clearly recovered the 3 genetic groups described in previous studies (Nicolas et al. 2016), reflecting both usage (Table *vs* Wine) and geographic origin (East *vs* West). However, we noted that the group assignment of some varieties may have been misclassified in previous analyses. This is likely due to the limited power of classification based on the restricted number of molecular markers used in previous analyses (Laucou et al. 2018; Fig. 5; Supplementary Fig. 8).

We then conducted GWAS on 3 traits related to grapevine production and wine quality (mean berry weight, shikimate concentration, and berry maturity date) using 2 genotypic matrices. The first matrix, based on a single linear reference genome, comprised ∼1.6 million biallelic SNPs and small indels (≤50 bp) (Sarah et al. 2025) (referred to as the SNPs-with-small-indels matrix hereafter). The second matrix, derived from the pangenome graph (described in the paragraph above), comprised ∼1.2 million biallelic variants, including not only SNPs but also biallelic small indels and large SVs (>50 pb) (referred to as the SNPs-with-small-and-large-SVs matrix hereafter). It is important to note that this matrix of ∼1.2 million variants incorporate a broader spectrum of genomic variants than the genotypic matrix obtained from the PN40024 reference (notably large SVs). We performed GWAS using the Bayesian sparse linear mixed model (BSLMM) implemented in GEMMA. (v.0.98.5; Zhou, Carbonetto and Stephens 2013), which allows a simultaneous capture of polygenic and major effect loci while accounting for linkage disequilibrium (LD hereafter).

GWAS with the SNPs-with-small-and-large-SVs matrix seemed to provide a better characterization of the genetic basis and architecture of the 3 agronomic traits compared to GWAS performed with the SNPs-with-small-indels matrix. This improvement is consistent with the inclusion of additional types of variants in the pangenome graph matrix, notably large structural variants (>50 bp) (Supplementary Tables 10 to 13). Specifically, analyses with the SNPs-with-small-and-large-SVs matrix allowed us to explain a similar or higher proportion of phenotypic variance for our 3 traits than analyses with the SNPs-with-small-indels matrix. For berry weight, the proportion of variance explained was similarly high between both approaches (PVE: 0.98 *vs* 0.99), with a similar proportion of phenotypic variance explained by loci with major effects (PVExPGE: 0.96 *vs* 0.97; Supplementary Table 13a). For maturity date, a higher proportion of phenotypic variance was explained with the SNPs-with-small-and-large-SVs matrix (PVE: 0.76 *vs* 0.71), with a higher proportion of phenotypic variance explained by major effect loci (PVExPGE: 0.71 *vs* 0.68; Supplementary Table 13b). Finally, for shikimate content, the proportion of phenotypic variance explained by major effect loci was higher with the SNPs-with-small-and-large-SVs matrix (PVExPGE: 0.96 vs 0.76; Supplementary Table 13c).

**Fig. 6.**
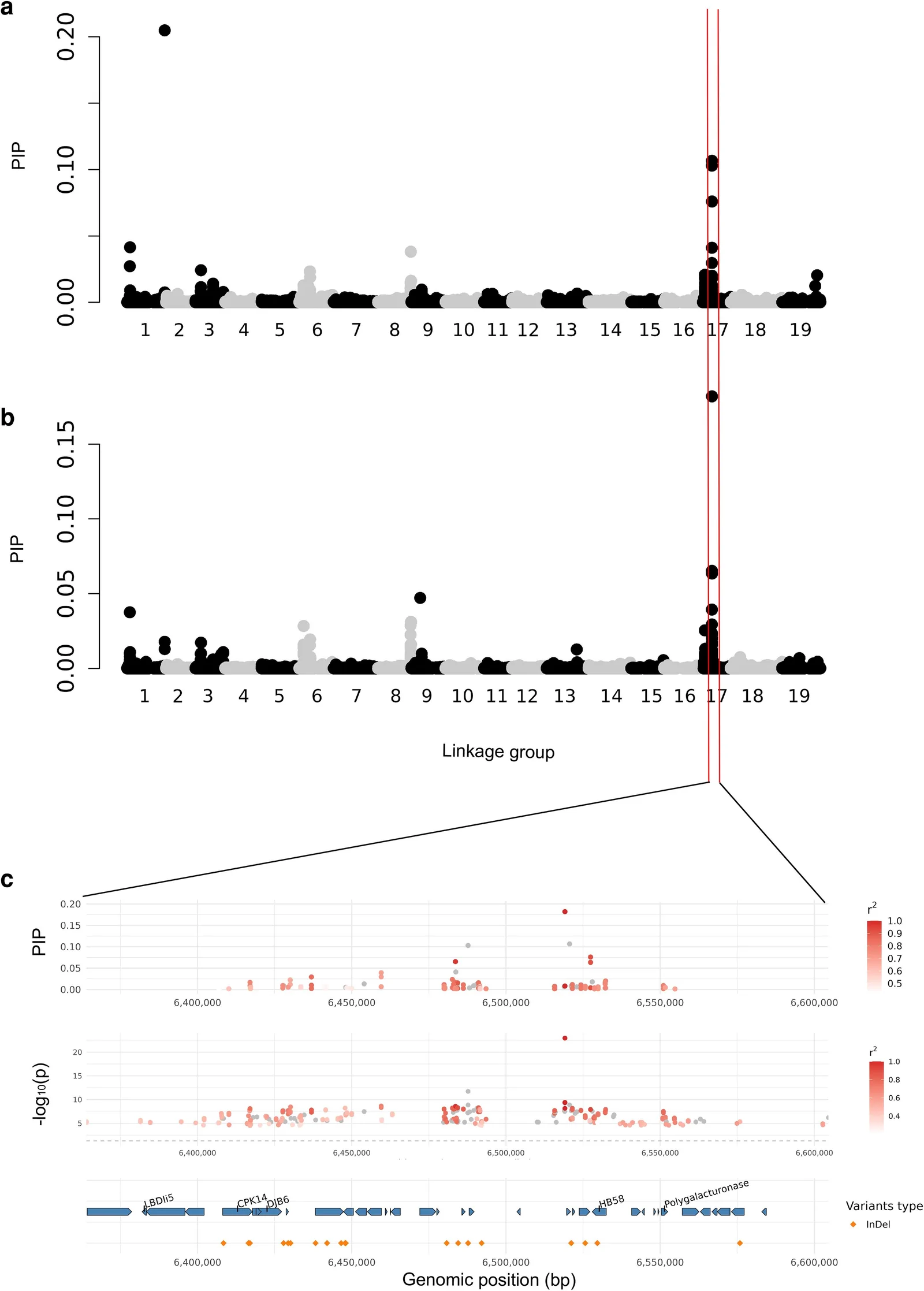
GWAS results for berry weight. Results of GWAS for berry weight (MBW), obtained when using the PN40024 as reference (a) or the pangenome graph as reference (b). Both Manhattan plots are projected onto the PN40024 reference. Both graphs show the posterior inclusion probabilities (PIP) estimated using gemma's BSLMM model. c) Zoomed view of the major effect locus detected on chromosome 17. The upper panel shows the BSLMM (PIP) results, colored according to linkage disequilibrium (LD with the main marker). The middle panel shows the results obtained with the GLM, MLM and FarmCPU models. The bottom panel shows the genomic annotation of the region, including the distribution of genes and indel, with the main candidate genes involved in berry development and ripening.

We then compared the number and identities of the major effect loci detected when using both matrices. For all traits, multiple loci are detected by both approaches, but with a higher number of loci identified using the SNPs-with-small-and-large-SVs matrix. For berry weight, we identified 239 major effect markers with the SNPs-with-small-and-large-SVs matrix, compared to 178 for the SNPs-with-small-indels matrix. We notably obtained narrower and clean association signals when using the SNPs-with-small-and-large-SVs matrix, particularly on chromosome 1, 8 and 17 (Fig. 6a and 6b, Supplementary Table 10). For maturity date, the number of major effect markers is slightly higher when using the SNPs-with-small-and-large-SVs matrix (276 vs 271) and the associated regions are slightly better defined (Supplementary Tables 12). For shikimate content, the number of major effect markers remains the same (21), but the association signals are narrower when using the SNPs-with-small-and-large-SVs matrix (Supplementary Table 11; Supplementary Figs. 9 and 10).

Interestingly, when using the SNPs-with-small-and-large-SVs matrix, we identified 32 major effect loci that we did not detect when using the SNPs-with-small-indels matrix (Table 3 and Supplementary Table 14). Notably, we detected a locus on chromosome 17, associated with berry weight (Fig. 6; Supplementary Figs. 11 and 12) and maturity date (Supplementary Figs. 13 and 14), which suggests that this locus is associated with both traits. This locus harbors several variants, including SNPs and small indels (<50 bp), as well as several previously annotated genes involved in berry development and ripening, such as: VvLBDli5 (Vitvi05_01chr17g07800), a lateral organ boundaries domain transcription factor differentially expressed during berry development; and VvHB58 (Vitvi05_01chr17g08020), an homeobox gene involved in fruit and seed development (Grimplet et al. 2017; Li et al. 2019) (Supplementary Table 15). We annotated all these variants using SnpEff (Cingolani et al. 2012) in order to explore their potential functional impact (Supplementary Table 15). We did not identify any high-impact variants directly affecting the LBDli5 or HB58 genes. We however identified 3 SNPs predicted to have a moderate impact on the Vitvi05_01chr17g08070 gene, which encodes for polygalacturonase, an enzyme involved in cell wall remodeling and softening of the berry skin during ripening (Deytieux-Belleau et al. 2008). We performed a LD analysis in the locus (Fig. 6c) and found that these variants showed a relatively high LD with the top marker of the GWAS signal (*r*^2^ = 0.7), suggesting that they could contribute to the observed association signal, either directly or through LD with the causal variant.

**Table 3.** Number of loci associated with berry weight, shikimate concentration and maturity date using the pangenome graph or the PN40024 genome as a reference^a^.

	Shared loci	Pangenome-specific loci	PN40024-specific loci
MBW	8	8	4
SHIKIMAT	28	14	11
SAMPLDAY	2	10	0
total	38	32	15

^a^Details on these loci are provided in Supplementary Table 13.

## Discussion

In this study we analyzed the genome assemblies of 15 cultivated grapevine varieties, combining comparative analyses on assembled pseudohaplotypes to study variation in gene content and transposable elements across variety. We also constructed a pangenome graph to study the variation in sequence across accession and evaluated the usefulness of genotyping an association panel from this graph to perform GWAS analyses. Our results are in accordance with previous pangenomic studies conducted in grapevine (Cochetel et al. 2023; Liu et al. 2024; Guo et al. 2025) and in other plant and animal species (Soybean: Liu et al. 2020; Strawberry: Qiao et al. 2021; Human: Liao et al. 2023; Pearl millet: Yan et al. 2023; Barley: Jayakodi et al. 2024; *Arabidopsis thaliana*: Lian et al. 2024).

Surprisingly for a plant with many varieties that were propagated clonally for millennia (Bouby et al. 2013; Xiao et al. 2025), we found well-conserved collinearity across the genomes of all the analyzed varieties, indicating that the cultivated grapevine retained a well-conserved genomic organization despite substantial diversification in agricultural practices, phenotype and usage. This result is certainly explained by the relatively short evolutionary history of the cultivated grapevine (∼11,000 years of domestication; Dong et al. 2023). Nonetheless, similar patterns of conserved collinearity have been reported in other wild and domesticated species (Yan et al. 2023; Lian et al. 2024), suggesting that massive genome reorganization (in terms of collinearity) does not necessarily play a major role in phenotypic and ecological diversification as was hypothesized by some authors (The uses of heresy by Stephen Jay Gould 1982; Goldschmidt 1982).

Despite this well-conserved collinearity, we identified substantial variation in gene content across varieties. Among the 55,662 orthologous genes detected, 44.7% are shared across all varieties and constitute the core gene set, whereas 55.3% are not shared by all varieties. Comparable proportions have been reported in other grapevine pangenomes, although estimates vary depending on sampling depth and methodological choices (Cochetel et al. 2023; Liu et al. 2024; Guo et al. 2025). Similar proportions have also been observed in multiple plant and animal species (Liu et al. 2020; Qiao et al. 2021; Liao et al. 2023; Yan et al. 2023; Jayakodi et al. 2024; Lian et al. 2024). This proportion is high but likely overestimated, both in our study and in previous work, given the challenges of accurately annotating functionally active genes. A substantial fraction of variable genes is likely nonfunctional and may correspond to pseudogenes or chimeric genes, while another fraction may represent duplicated or deleted genes with near-neutral effects. Such variable genes are likely maintained in populations under a mutation-drift equilibrium. Still, a significant fraction of variable genes may be associated with adaptation to biotic and abiotic constraints. Functional enrichment analyses show that variable genes are enriched for biological functions related to secondary metabolism, responses to biotic and abiotic stresses, and environmental adaptation. Enrichment for similar functions has been reported in *Arabidopsis thaliana* (Kang et al. 2023), *Panicum miliaceum L.* (Chen et al. 2023) and grapevine super-pangenomes (Cochetel et al. 2023; Guo et al. 2025), supporting the view that variable genes constitute an important reservoir of genetic diversity for phenotypic and ecological diversification.

In this context, gene structure may also contribute to gene stability within pangenomes, without necessarily being the primary determinant. Variable genes appear to be enriched in pseudogenes and almost neutral genes, which may be subject to lower selective pressures (Li et al. 2014). Under this hypothesis, these genes may be approaching a mutation-drift equilibrium, which could explain their greater tendency toward presence/absence variations and proximity of structural rearrangements (Li et al. 2014). Conversely, genes with a more restricted function may be more conserved across varieties. As observed in other species, notably *Prunus mume* (Huang et al. 2026) and *Brassica aleracea* (Golicz et al. 2016), variable genes exhibit reduced length and lower number of exons. While these features are consistent with reduced evolutionary constraint, they do not in themselves support a role of gene structure. Rather, they may reflect the enrichment in pseudogenes and almost neutral genes described above. Taken together, gene structure and functional status may jointly influence the likelihood of genomic variation, although their respective contributions cannot be clearly distinguished here.

A comparison of our results with the cultivated grapevine pangene set from Lui and colleagues (Liu et al. 2024) showed that, although the core gene set is largely conserved between the 2 pangene sets, the variable gene category appears to be significantly more sensitive to annotation methods. The differences observed in gene detection and classification, as well as the numerous changes in pangenome categories (core, soft-core, and variable genes), indicate that the assignment of presence/absence statuses is strongly influenced by methodological choices, although biological variations also contribute to the observed differences. More generally, the literature emphasizes that the representativeness of a pangenome depends on the diversity and structure of the sample, particularly when characterizing the variable fraction of the pangenome (Jayakodi et al. 2024). Overall, these findings suggest that comparisons between independently constructed pangene sets should be interpreted with caution, particularly for the variable gene set, whose definition appears less stable than that of the core gene set. Altogether, these results suggests that current core and variable gene set descriptions should be taken with caution and needs replication with more input genomes and standardized annotation practices.

TEs, through the diversity of their transposition mechanism and evolutionary dynamics, constitute a major source of a genetic variation, producing insertions, deletions and rearrangements that have been shown to contribute to the genetic diversification of closely related genomes (Benjak et al. 2008; Carrier et al. 2012; Chuong et al. 2017; Foria et al. 2020). Our results reinforce the idea that TEs are major drivers of recent structural variation in genomes. Indeed, in our study, class I TEs dominate the TE landscape. Their copy-and-paste transposition mechanism likely contributes to their long-term accumulation in conserved genomic compartments (Piegu et al. 2006; Cochetel et al. 2023). This prevalence, already observed in grapevines and other plants, supports the idea that retrotransposons play a major role in the long-term structuring and expansion of plant genome, and more specifically grapevines. In contrast, class II DNA transposons, which are mobilized using a cut-and-paste mechanism, are more frequently associated with variable regions of the genome. It has been shown that the “cut and paste” transposons of grapevine have transposed and amplified genomic sequences, some of them being “domesticated” and probably assuming new biological functions (Benjak et al. 2008). Comparable patterns have been observed in other plant genomes like *Arabidopsis thaliana* (Kang et al. 2023). Furthermore, our results suggest that a significant portion of the variation in gene content observed in our study is certainly linked to the dynamics of TEs. By favoring the emergence of structural mutation, they favor the appearance of gene duplication, gene loss and contribute greatly to the evolution of the variable part of the genome (Lisch 2013). Beyond their structural impact, TEs are also preferentially associated with specific functional categories of genes. Our GO enrichment analysis revealed that TEs are overrepresented near genes involved in translation, metabolism, development, stress response, and intracellular transport. This distribution suggests either selective constraints or a preferential retention of insertions with regulatory effects, which could influence several biological pathways in grapevine. Enrichment near genes related to translation and ribosome biogenesis could modulate the efficiency of protein synthesis, potentially affecting plant growth and development. Proximity to genes involved in secondary metabolism, including flavonoid and carbohydrate pathways, could contribute to variation in fruit composition and stress resilience. Similarly, the presence of TEs near genes associated with meristem activity, leaf and root development, or hormonal signaling could underlie phenotypic plasticity in traits such as bud break, flowering, and fruit ripening (Raingeval et al. 2024). Overall, these observations support a model in which TEs shape both genome structure and functional potential, providing a mechanistic link between structural variants, gene regulation, and phenotypic and diversity (Lisch 2013; Chuong et al. 2017). We observed differences in the distribution of frequencies of genes and TEs in our comparative studies. These differences should, however, be interpreted with caution, as these 2 classes were not defined at the same level of organization. Genes were analyzed at the level of orthologous families, whereas transposable elements were grouped into families based on annotations combining structural and homology-based approaches (panTE families). Furthermore, transposable elements identified solely by structural criteria and lacking family assignment, often labeled by genomic coordinates, are excluded from library construction and clustering (following panEDTA recommendations), resulting in a frequency distribution with fewer low-frequency elements.

A significant proportion of the sequence present within our pangenome graph is absent from the PN40024 reference genome, highlighting the potential limitations of using a single reference genome to describe and characterize a substantial amount of the genetic diversity present within a species. However, these sequences included in the pangenome graph remain characterizable via variant calling from the graph. Performing GWAS using a pangenome graph as a genotyping reference could therefore be a very useful tool to better characterize the genetic basis of important agronomic and evolutionary traits (Du et al. 2025). Our results show that GWAS using the pangenome graph as a reference produce different results from those obtained with a single reference genome (in our case the PN40024 v5 reference), notably with a higher proportion of phenotypic variance explained by genetic markers for the pangenome graph approach. In particular, we identified 32 loci associated with our traits that we did not detect with the single reference genome approach. These differences were observed despite a comparable number of markers used as input for both approaches, suggesting that the observed gain in explained phenotypic variance primarily comes from the inclusion of large structural variants (>50 bp) in the GWAS analyses (Chakraborty et al. 2019; Bai et al. 2026). Several of these associated loci were consistently identified by different GWAS models (GLM, MLM, FarmCPU and BSLMM), which suggests that these associations are largely independent of the statistical model used, thereby reducing the risk of false positives. Future analyses should nonetheless include replication in an independent panel as well as functional validation of these newly identified loci to make sure they are not false positives. Overall, our results align with results found in other cultivated species, such as tomato, millet, and cotton (Zhou et al. 2022; Chen et al. 2023; Meng et al. 2025), where a pangenome approach enabled the detection of new loci (Hirsch et al. 2014; Qin et al. 2021). A comparison of our GWAS results with published studies indicates that 12 loci we identified were also identified in previous studies, supporting the robustness of our results (Delfino et al. 2019; Flutre et al. 2022; Liu et al. 2024; Supplementary Table 11). Notably, the locus identified on chromosome 17 overlaps with a locus previously described in a biparental population for a related trait (Delfino et al. 2019), suggesting the presence of a consistent genetic signal in this genomic region across different mapping panels. Furthermore, this region is located near a locus associated with berry width in Grapepan.V1 (Liu et al. 2024), although the precise definition of the traits differs between studies (berry width vs berry weight), which limits a direct comparison and must be taken into account in the interpretation.

Some methodological limitations to our work should nonetheless be discussed. One limitation concerns the lack of long-range phasing data, such as Hi-C data or high-resolution genetic maps, to perform genome assembly in this study. Because this data is lacking, the pseudohaplotypes used in this study may contain haplotype switches, leading to locally chimeric sequences. However, despite the potential presence of these switches, Merqury analyses indicate that the assemblies capture almost all genomic kmers, suggesting that the majority of sequence variation is well represented. Consequently, the pangenome graph should contain a set of variants similar to that which would be obtained with perfectly phased assemblies. However, the impact of this potential bias is likely limited in our study, due to the overall structure of the pangenome graph we observed (high collinearity) and the predominance of small variants over large variants. Indeed, phasing errors should mainly affect long-range haplotype structures rather than local variations such as SNPs and small indels. A second limitation concerns multiallelic variants. Indeed, a significant proportion of the structural variants we identified are multiallelic, and cannot be used in a straightforward manner in GWAS, as models use biallelic markers.

The process of constructing and using a pangenome graph remains very expensive computationally, which may restrict its use in the near future to species with relatively small genomes. For such species, SV imputation from SNP array or short-read data (Bai et al. 2026) or pairwise whole-genome alignment (Chakraborty et al. 2019) could represent powerful and cost-effective alternatives to developing a pangenome graph (eg 10.1038/s41467-019-12884-1). In any case, the development of methods that handle multiallelic variants, and optimize computational time are sure to benefit future research in pangenomic and would enable us to fully exploit the rich data provided by pangenomes.

## Conclusion

Pangenomes provide access to a substantial reservoir of dispensable genes and sequences that may be missed when using a single reference genome, a significant fraction of which is likely involved in adaptation and phenotypic diversification. Despite recent advances in characterizing plant pangenomes, our results indicate that current genes classification into core and dispensable genes should be taken with caution. The use of pangenome graphs in GWAS, because they facilitate the inclusion of large structural variants into the analyses, may improve the characterization of the genetic architecture of agronomic traits. Ultimately, pangenomic analyses may help better characterize the genetic basis of traits and help refine current hypotheses about the genetic bases of adaptation. They may also benefit the breeding of plant and animal varieties better suited to future challenges, notably climate change.

## Supplementary Material

jkag168_Supplementary_Data

## Data Availability

Data were deposited in NCBI under the following BioProject accession numbers: PRJEB100755 (PacBio data), PRJEB95058 (illumina short read data), and PRJEB107006 (RNA-seq data). Genome assemblies and annotations (genes and transposable elements) are available at https://doi.org/10.57745/CKD4H5. Scripts are available at https://forge.inrae.fr/baptiste.pierre/pangenomics-analysis. Supplemental material available at G3 online.
