# Supplementary material for: Pangenomic analyses in the cultivated grapevine confirm high genomic collinearity and extensive dispensable gene content likely involved in adaptation
Source: G3 (Bethesda). 2026 Jul 9;16(9):jkag168. doi: 10.1093/g3journal/jkag168 (PMC13535373; doi:10.1093/g3journal/jkag168)
Supplement: Supplementary file 1 [file jkag168_supplementary_data.zip › Supplementary_Figures_G3-2026-406972.docx]

**Supplementary Information for**

Pangenomic analyses in the cultivated grapevine confirm high genomic collinearity and extensive dispensable gene content likely involved in adaptation

B. Pierre *et al.*

Corresponding authors: Romain Villoutreix (romain.villoutreix@inrae.fr - phone: +33 4 67 61 71 21) & Gautier Sarah ([gautier.sarah@inrae.fr](mailto:gautier.sarah@inrae.fr)- phone: +33 4 67 61 56 51). Address: 389 Av. Agropolis, 34060 Montpellier, France.

**The file includes:**

**Fig. S1 to S14**

All scripts used in this study are publicly available at: <https://forge.inrae.fr/baptiste.pierre/pangenomics-analysis>

The data is available at the following link: <https://doi.org/10.57745/CKD4H5>

**Figure S1. Distribution of kmer multiplicity in different grapevine varieties.**

Each panel (A-I) represents a variety or set of varieties compared to two sets of pseudohaplotypes purged from puge-dup tools. The x-axis indicates kmer multiplicity, while the y-axis indicates the number of kmers corresponding to each multiplicity. The colors represent the classification of kmers: grey (ready-only) kmer present only in the reads, absent from the purged haplotypes; red (hap1.purged-only) kmers present only in the first purged haplotype; blue (hap2.purged-only) kmers present only in the second purged haplotype; green (shared) kmers shared between the two purged haplotypes.

Panels: A: ‘Alexandroouli’; B: ‘Blank blauer’ C: ‘Bou khanzir noir’; D: ‘Camaraou noir’; E: ‘Corbeau’; F: ‘Morrastel’; G: ‘Opsimo Edessis’; H: ‘Pagadebiti’; I: ‘Riminese’;

This figure illustrates the distribution of haplotype-specific kmers relative to shared kmers, allowing for the evaluation of the complementarity and genomic diversity captured by the purged pseudohaplotypes.

**Figure S2. Haplotype-by-haplotype synteny analysis between the PN40024 reference and 15 grapevine varieties for chromosomes 2 to 19 (a-r).** Complete chromosome alignments were performed using SyRi and visualized with plotter. Syntenic regions are shown in grey, while different classes of structural variations are color-coded, including inversions, duplications, translocations, insertions and deletions. Assembly gaps are also indicated. Overall, chromosome organization is largely conserved between accessions with occasional structural variation to specific regions of the chromosomes. Stars indicate newly generated assemblies. MR: ‘Morrastel’, CN: ‘Camaraou noir’, CH: ‘Chasselas’, CO: ‘Corbeau’, PN: ‘Pinot noir’, PNT2T: PN40024, MH: ‘Muscat hamburg’, UB: ‘Ugni blanc’, RI: ’Riminese’, PA: ‘Pagadebiti’, BB: ‘Blank blauer’, OE: ‘Opsimo edessis’, BKN: ‘Bou Khanzir noir’, AL: ‘Alexandroouli’, TS: ‘Sultanina’, MF: ‘Manicure finger’.

**Figure S3. Gene ontology (GO) enrichment analyses for soft-core genes.** The bubble chart shows the significantly enriched biological terms, with the x-axis representing the ratio of enriched genes over the total number of genes (GeneRatio), the size of the bubbles representing the number of enriched genes and colors representing the level of significance for the enrichment (p.adjust)

**Figure S4. Analysis of gene overlaps between pangene sets.** Visualization of gene overlaps between different studies using UpSet plot. The top bars indicate the number of genes shared between combinations of datasets, while the connected dots represent the sets under consideration. The total sizes of the sets are indicated on the bottom axis.

**Figure S5. Comparison of gene classifications between pangenome studies.** Comparison of gene categories (core, soft-core, variable and absent) between our pangene set and Grapepan (Liu *et al.* 2024). The stacked bars represent the classification of genes in one dataset and their corresponding status in the other (Source → Target). The left and right panels show, respectively, the transitions from our pangene set to Grapepan’s and from Grapepan’s to our pangene set. This figure illustrates the concordances and transfers between categories, highlighting the impact of differences in annotation and pangene construction on gene classification.

**Figure S6. Density of large structural variants (>50bp) in the cultivated grapevine genome**. The heat map shows the density of large SVs (>50bp) detected along the 19 chromosomes of *V.vinifera*, calculated for 30kb windows. The colors indicate the number of SVs per window, from green (low density) to red (high density). This is represented on the PN40024 reference genome.

**Figure S7. Overlap of structural variants and single nucleotide polymorphisms in the cultivated grapevine genome.** Joint distribution of SVs and SNPs along the 19 chromosomes of the cultivated grapevine calculated for 30 kb windows. Green regions indicate windows containing both SVs and SNPs (intersection), red regions indicate windows containing only SNPs, and blue regions indicate windows containing only SVs. This representation highlights the complementarity of the two types of markers in covering the entirety of the genome. The grey area corresponds to genomic windows with no detected SVs and SNPs or with missing data. This is represented on the PN40024 reference genome.

**Figure S8**. **Variance explained by principal components.** Barplot showing the proportion of variance explained by each principal component in the PCA based on markers genotyped using the pangenome graph.

**Figure S9.** **Manhattan and Q-Q plots for SHIKIMATE using the PN40024 reference.** (a) Manhattan plot showing genome-wide association signals. This is represented on the PN40024 reference genome. (b) Corresponding Q-Q plot of observed vs expected -log10(p-values). For each GWAS three methods are superimposed: FarmCPU (dark blue), MLM (yellow) and GLM (light blue).

**Figure S10.** **Manhattan and Q-Q plots for SHIKIMATE using the pangenome graph as a reference.** (a) Manhattan plot showing genome-wide association signals. This is represented on the PN40024 reference genome. (b) Corresponding Q-Q plot of observed vs expected -log10(p-values). For each GWAS three methods are superimposed: FarmCPU (dark blue), MLM (yellow) and GLM (light blue).

**Figure S11.** **Manhattan and Q-Q plots for MBW using the PN40024 reference.** (a) Manhattan plot showing genome-wide association signals. This is represented on the PN40024 reference genome. (b) Corresponding Q-Q plot of observed vs expected -log10(p-values). For each GWAS three methods are superimposed: FarmCPU (dark blue), MLM (yellow) and GLM (light blue).

**Figure S12.** **Manhattan and Q-Q plots for MBW using the pangenome graph as a reference.** (a) Manhattan plot showing genome-wide association signals. This is represented on the PN40024 reference genome. (b) Corresponding Q-Q plot of observed vs expected -log10(p-values). For each GWAS three methods are superimposed: FarmCPU (dark blue), MLM (yellow) and GLM (light blue).

**Figure S13.** **Manhattan and Q-Q plots for SAMPLDAY using the PN40024 reference.** (a) Manhattan plot showing genome-wide association signals. This is represented on the PN40024 reference genome. (b) Corresponding Q-Q plot of observed vs expected -log10(p-values). For each GWAS three methods are superimposed: FarmCPU (dark blue), MLM (yellow) and GLM (light blue).

**Figure S14.** **Manhattan and Q-Q plots for SAMPLDAY using the pangenome graph as a reference.** (a) Manhattan plot showing genome-wide association signals. This is represented on the PN40024 reference genome. (b) Corresponding Q-Q plot of observed vs expected -log10(p-values). For each GWAS three methods are superimposed: FarmCPU (dark blue), MLM (yellow) and GLM (light blue).
